# Internal connectivity of the mouse mesocortical ring and functional implications

**DOI:** 10.1007/s00429-026-03110-y

**Published:** 2026-05-08

**Authors:** Luis Puelles, Elena Garcia-Calero

**Affiliations:** 1 Departamento de Anatomía Humana y Psicobiología, Facultad de Medicina, Universidad de Murcia, Campus de Ciencias de la Salud, Murcia, 30120 España; 2 Instituto Murciano de Investigación Biosanitaria Pascual Parrilla (IMIB), Murcia, 30120 España

**Keywords:** Limbic system, Connectome, Posterior orbitary cortex, *Self*, Psychopathy

## Abstract

The mammalian mesocortex (MCx) was redefined recently as a complete ring intercalated between the allo- and iso-cortex, attending to the consistent expression of 46 selective gene markers. The MCx exhibits various other characteristics such as low myelin content of its fibers, a property directly related to its function and susceptibility to degenerative disorders. Irrespective of its shared molecular profile, the MCx ring differentiates into different sectors with singular molecular and cytoarchitectonic characteristics, such as the posterior orbitary cortex, the cingulate cortex, and the insula. In the present study, using anterograde connectivity experiments published in an Allen Institute for Brain Science public database (https://brain-map.org/our-research/connectivity), we analyzed the internal connectivity of the mesocortical ring. We observed that its different sectors are multiply interconnected, possibly achieving integrative functions. The medial posterior orbitary cortex stands out; it appears to play a higher hierarchical role within the MCx ring by sending and receiving projections from all mesocortical sectors.

**Supplementary Information:**

The online version contains supplementary material available at 10.1007/s00429-026-03110-y.

## Introduction

The concept of a mammalian mesolimbic ring with a characteristic cytoarchitectonic and molecular profile was defended recently by Puelles et al. (2019, 2024). The notion of a “limbic” cortex is not new (Broca 1878), but early concepts mixed the hippocampal and cingulate cortex using a positional definition of the term “limbic”, implying arrangement along the medial edge (limbus) of the cortex, rather than a particular structure and function. Indeed, the hippocampus is involved in generation of episodic memory, while the cingulate cortex processes emotions, and their respective cytoarchitectonic, connective and molecular profiles are quite different (Rolls 2014, 2015; Yao et al. 2023). The current idea of the limbic cortical system implies it functionally in the subjective evaluation of experience and behavior plans (i.e., emotions, motivation). The present mesolimbic ring concept arises partly from the ‘intermediate areas’ in the cortical model of Filimonoff (1947), and an earlier idea of Rose (1926) of cytoarchitectonically differentiated 5-layered transitional cortex separating 3-layered allocortex from 6-layered isocortex. This initially incomplete concept was later variously developed by other authors (including pioneering molecularly based work such as Pattabiraman et al. 2014 and Puelles et al. 2019; review in Puelles et al. 2024). The modern ‘molecular’ concept is consistent with the present developmental knowledge about multiple early morphogen sources peripheral to the cortical field whose diffusing signals seem to pattern the mammalian cortex into concentric allocortical and mesocortical rings around an inner isocortical island (e.g., Borello and Pierani 2010; Puelles 2011; Puelles et al. 2019, 2024). There is abundant evidence that cingulate, insular and orbitofrontal cortical areas – now listed as integral parts of the mesocortical ring- are strongly involved in emotional mechanisms (Rolls 2014, 2015). Other mesocortical sectors, such as the perirhinal and postrhinal areas are less clearly related to emotions. The classical authors (notably Rose 1926) included the retrosplenial cortex in the mesocortical category, though this conclusion was contradicted by the marker analysis of Puelles et al. (2024), whose results showed that it did not express any of the 46 MCx markers, was well-myelinated, and shared some markers with parahippocampal cortex domains (Yao et al. 2023); there are also physiological data suggesting a parahippocampal profile for that area. Contrarily, Puelles et al. (2024) identified a molecular mesocortical typology of two novel cortical areas running parallel to the retrosplenial cortex, medially to the visual cortex, which they named postsplenial and parasplenial areas. These actually close the modern mesocortical ring between the postrhinal and the cingulate areas. These studies therefore crystallize into the notion that the mesolimbic cortical ring, which actually was revealed to be 6-layered rather than 5-layered, possibly evolved as a variant of the central isocortical island whose singular differential molecular profile may have adapted functionally as a whole for cortical subjective (emotional) evaluation of sensations and action plans (Puelles et al. 2024).

As noted above, the molecularly defined mesolimbic cortex (Puelles et al. 2024) is tentatively divided into several sectors whose hypothetic differential properties are still poorly known, in terms of both differential structure and molecular characteristics. If we begin to list them rostrally, we start with the posterior orbitary cortex (POrb), which we subdivide practically into medial and ventral-lateral posterior orbitary areas (POrbM, POrbVL). The POrb is continuous laterally with the insula and peri- and postrhinal regions (Ins; PeRh; PoRh; the ectorhinal cortex -EcRh- seems to extend dorsally the PeRh area). The PoRh area frames caudally the visual cortex, dorsally to the parasubicular and entorhinal cortical (parahippocampal) areas and ends as it reaches the medial hemispheric wall. Here it connects with the novel postsplenial (PoSp) area, a narrow, wedge-shaped mesocortical domain lying just behind the retrosplenial cortex (ReSp). Proceeding now rostralward, once the ReSp cortex appears in the sections, the PoSp area is substituted by the parasplenial area (PaSp) sector, which extends rostralward laterally to the whole length of the retrosplenial cortex, placed medially to successive visual, somesthetic, and motor associative areas, until it meets rostrally the caudal end of the cingulate mesocortical sector after the retrosplenial cortex ends (Cing; various Cing subareas are traditionally distinguished). The PaSp area lies medially at the convexity of the cortex, while the Cing is located at the interhemispheric face. Approximately at the level of the knee of the corpus callosum, the Cing cortex transits into the likewise mesocortical prelimbic cortex (PL), which in turn connects with the medial POrb area, thus closing the ring.

In terms of cytoarchitecture, these diverse mesocortical sectors share some cytoarchitectonic properties, particularly regarding the relative thickness and depth of the 6 layers that are uniformly present (as detected with layer-specific gene markers; Puelles et al. 2024). The layer I is well developed. The layers II-III-IV are on the whole thinner than in the isocortex, so that the layer V distinctly lies closer to the brain surface. The classical authors described in the insular and cingulate sectors of the ring ventrodorsal incremental degrees of complexity representing transitional periallocortical into proisocortical cytoarchitectonic steps also know alternatively as agranular, dysgranular and granular subdivisions. These changes also are partly present in the PeRh-EcRh domains. Granularity refers to the variable amount of layer IV components.

Regarding the connectivity of the mesocortical ring, the most relevant works that have attended to this topic correspond to the group of Helen Barbas, whose “structural model of the cortex” attends to areal changes in the connectivity patterns (Barbas 2015; GarcDeadíGarcía-Cabezas et al. 2019). In this model, cortical connectivity is described comparatively across the whole cortical field as following a hierarchical pattern determined by the laminar directionality of the connections. Due probably to the earlier misconception that the retrosplenial cortex was a part of the limbic mesocortex, a clear schema of an intrinsic connectivity between different parts of the mesocortical ring was not developed, nor was examined purposefully even by authors defending its theoretical existence. The literature nevertheless does register various relevant partial experimental data, usually interpreted outside of the ring concept. As a consequence of the novel evidence of a shared molecular pattern along the entire mesocortical ring, our curiosity was drawn to the question about the possibility that systematic internal connections occur *within* the mesocortical ring, considering specifically the updated list of MCx sectors described by Puelles et al. (2024). A collateral interest was to see whether any of the sectors of the ring was hodologically predominant over the others in such intrinsic connections, that is, the issue whether we can speak of a hierarchy in the internal connectivity of the limbic ring.

In this report, we concentrated in examining the relatively abundant Allen Institute data on mouse cortex connectivity experiments. We analyzed all available anterograde labeling injections that clearly fall into *our* mesocortical sectors according to the map published in Puelles et al. 2024 (i.e., irrespective how they were classified by the Allen technicians, that did not know about the molecularly defined MCx ring when these experiments were done). We primarily checked and mapped the details of connectivity of each mesocortical sector with neighboring or more distant ipsilateral or contralateral sectors of the mesocortical ring. Obviously, we also observed connections with various other telencephalic targets, which will be briefly mentioned.

We did find extensive evidence of systematic connections of each mesocortical sector with its nearest limbic ring neighbors in both directions and also reaching often more or less distant ring parts, depending on the sectors. We also identified the POrbM area as the mesocortical area that possibly ranks above the rest, due to its higher degree of afferent and efferent connectivity with all other ring portions.

## Materials and methods

### Allen Brain connectivity atlas

**Fig. 1:**
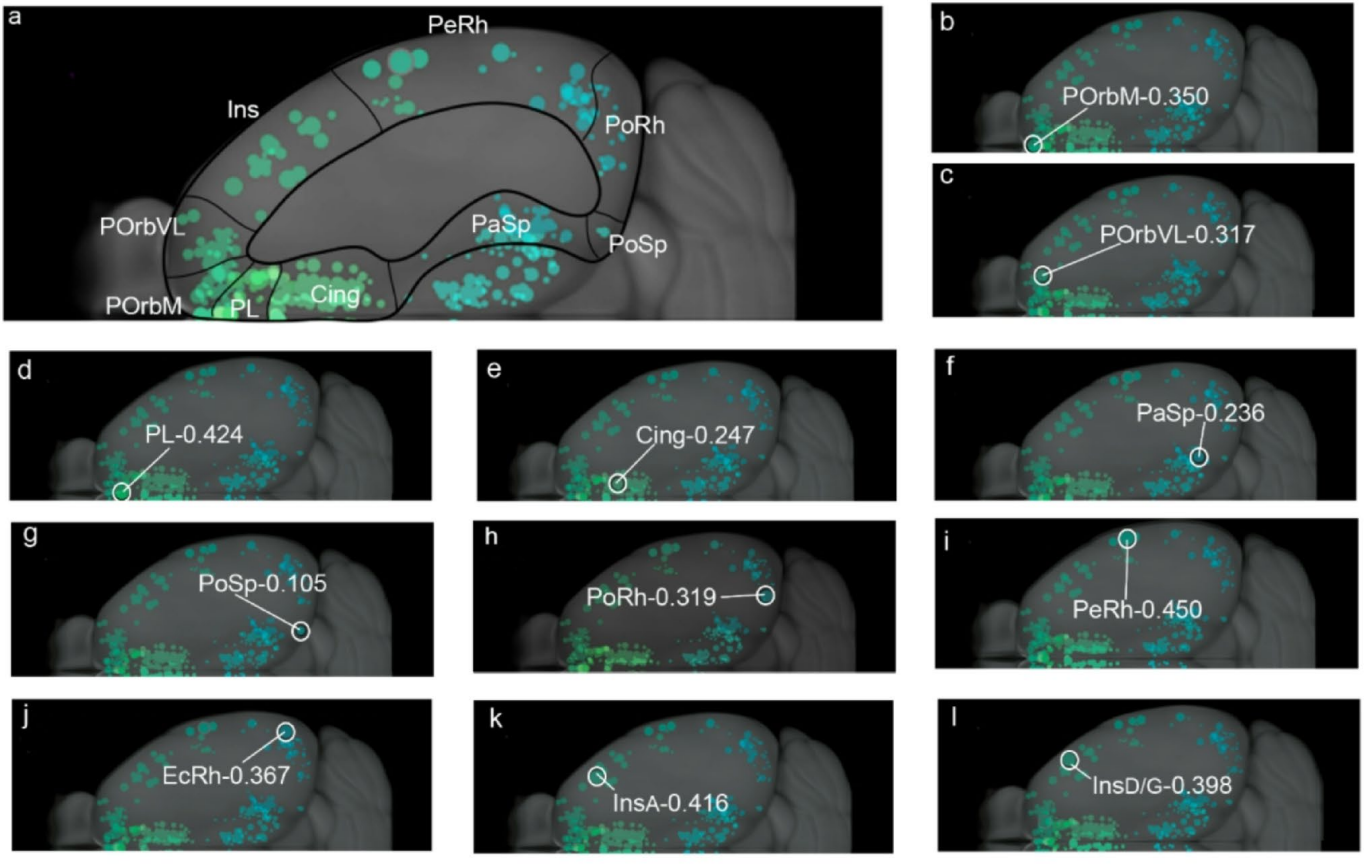
Injection sites in the mesocortical ring. Schema of selective virus injection sites in the molecularly defined mesocortical ring using data obtained from the Allen Brain database (https://brain-map.org/our-research/connectivity). For clarity, we combined all the relevant injection sites on the right brain half and to this end flipped the cases mapped on the left half of the telencephalon into the right half representation. **a** This image represents all the injection points in the dataset examined, framed within the mesocortical ring divided into the sectors postulated in Puelles et al. (2024). The schema also shows injections described in the Allen database as located in the ‘retrosplenial cortex’; it can be observed that the most lateral ones fall actually within our molecularly defined PaSp mesocortical sector, while the medial ones are properly extralimbic and retrosplenial. **b**-**l** Each image indicates the exact location of the representative virus injection described in the Results section (i.e., the specific case)

We used the Allen Brain Mouse Connectivity Atlas (ABMCA) to develop the present work (https://connectivity.brain-map.org/ ). To look for injection sites in the mesocortical ring following the Puelles et al. (2024) map we used the Allen source structures: VISC (visceral area), VISpl (posterolateral visual area), VISpm (posteromedial visual area), VISpor (postrhinal area), ACA (anterior cingulate area), PL (prelimbic area), ILA (infralimbic area), ORB (orbitary area), AI (agranular insular area), RSPagl (retrosplenial area, lateral agranular part) RSPv (retrosplenial area, ventral part) PERI (perirhinal area), ECT (ectorhinal area; Fig. 1a; dates 29/10/2025-05/02/2026). We chose cases with injection site volume between 0.200 and 0.500 mm^3^. After examining cases with all the different injection sizes, we concluded that only this range of injection volumes is adequate for our inquiry: smaller ones do not show the more distant projections, and the excessively large ones complicate interpretation by invading different neighboring sites (but see also Juárez-Leal et al. 2022). Table 1 shows the cases chosen from ABMCA followed by a *hyphen* and the injection site volume, for each mesocortical sector defined in Puelles et al. (2024).

**Table 1:** Injection experiments in the mesocortical ring

POrbM	POrbVL	PL	Cing	PaSp
ORBm-0.350	ORBl-0.263	PL-0.477	ACAd-0.239	RSpagl-0.308
ORBm-0.288	ORBl-0.219	PL-0.424	ACAd-0.205	RSPagl-0.275
ORBm-0.200	ORBl-0.423	PL-0.260	ACAd-0.247	RSPagl-0.223
ORBm-0.211	ORBl-0.232	PL-0.242	ACAd-0.274	VISpm-0.276
ORBm-0.283 (+ PL)	ORBl-0.317	PL-0.258	ACAd-0.432	VISpm-0.236
ORBvl-0.380 (+ PL)	ORBvl-0.296	PL-0.391	ACAd-0.227	VISpm-0.436
	ORBvl-0.202	ORBm-0.208	ACAv-0.222	
	ORBvl-0.423	ILA-0.484	ACAv-0.229	
	ORBvl-0.386	ILA-0.205	ACAv-0.392	
			ACAv-0.334	
			PL-0.211	
**PoSp**	**PoRh**	**PeRh**	**EcRh**	**Ins**
RSPagl-0.105	VISpl-0.319	VISC-0.448	VISpor-0.310	AId-0.398 gr/dis
	VISpor-0.400	VISC-0.450	VISpor-0.360	AId-0.473 gr/dis
		AIp-0.280	VISpor-0.367	AId-0.243 gr/dis
		AIp-0.245 (claustrum)	VISpor-0.212	AId-0.212 agr
		VISpor-0.355	VISpor-0.252	AIv-0.416 agr
				AIv-0.355 (claustrum)
				AIv-0.349 (claustrum)

Cases chosen from Allen Brain Mouse Connectivity Atlas (https://connectivity.brain-map.org/*).* The locus tag for each mesocortical sector defined in Puelles et al. (2024) is followed by a *hyphen* and the injection site volume in mm^3^ units

The injections indicated in the ABMCA were not always in the indicated site according to our criteria. Therefore, it was not exceptional to have to reclassify some injection sites. Once the cases have been reclassified, the final way to name them, for example in Fig. 1, is by using the name that corresponds to it according to our cortical ring model (POrbM, POrbVL, PL, Cing, PaSp, PoSp, PoRh, PeRh, Ect, Ins) plus the *hyphen* and the injection volume. For example, for the case listed as ORBm-0.208 by the Allen Institute (Table 1) we judged that the injection site lies in the prelimbic cortex rather than in POrbM (i.e., it lays rostral to the accumbens nucleus, in accordance with Paxinos and Franklin 2019), and, therefore, we did not study it under the POrbM category, but under PL (i.e., entered it as PL-0.208). Reclassified cases are indicated in the legend of the figures in Supplementary Information. In some injections (i.e., Cing injections: ACAd-0.300 and ACAd-0.412) the tracer did not diffuse though the location was correct; therefore, these cases were not considered.

The injections were represented on the right side of the telencephalon. For those cases where the injections were performed on the left side, we flipped the image horizontally in the coronal sections and vertically in the density maps (indicated in the legend of the corresponding figure). In this work we are interested in cortico-cortical connections (within the ring), but it needs to be cautioned that in many cases subcortical connections are also due to injection sites affecting the deep cortical layers.

### Image processing

The digital images were processed with Adobe Photoshop Elements and Adobe Illustrator (version CC) software (Adobe Systems MountainView, CA, USA).

## Results

We analyzed the anterograde projections from different sectors in the mesocortical ring as mapped out in Puelles et al. (2024), using the high-resolution connectivity data from ABMCA. Brief mention was made also of projections of each sector to the isocortex, allocortex and subpallium (cortico-amygdalar projections will be analyzed in a separate publication). Figure 1a shows a map of a reconstructed top view of the ring-shaped cortical (mouse) territory with all available tracer injections placed inside or next to the mesocortex ring. All of them were examined in detail, filtering out or reclassifying a number of them according to the updated definition of the mesocortex. The specific cases representative for the different ring sectors that were selected for description in Results are shown in Figs. 1b-l. In the following text we identify the individual cases by the abbreviation of the area of injection followed by a *hyphen* and the amount in cubic millimeters of virus solution injected according to ABMCA data (e.g., Orbm-0.350).

### Projections from the medial and lateral posterior orbitary cortex to regions of the mesocortical ring and other telencephalic areas

We first analyzed the anterograde connectivity experiments classified in the Allen database as placed in ‘medial orbitofrontal cortex’ (ORBm in ABMCA). This category refers to the area included in our *medial posterior orbitary cortex* (POrbM) (Puelles et al. 2024). We verified that all the listed Allen virus injections were in the correct area according to our map, eliminating any doubtful ones. After preliminary analysis, we selected for detailed analysis only the cases with injection volumes equal or higher than 0.200 and equal or lower than 0.500 mm^3^. Within these restrictions, we obtained 6 useful cases in POrbM (Table 1).

**Fig. 2:**
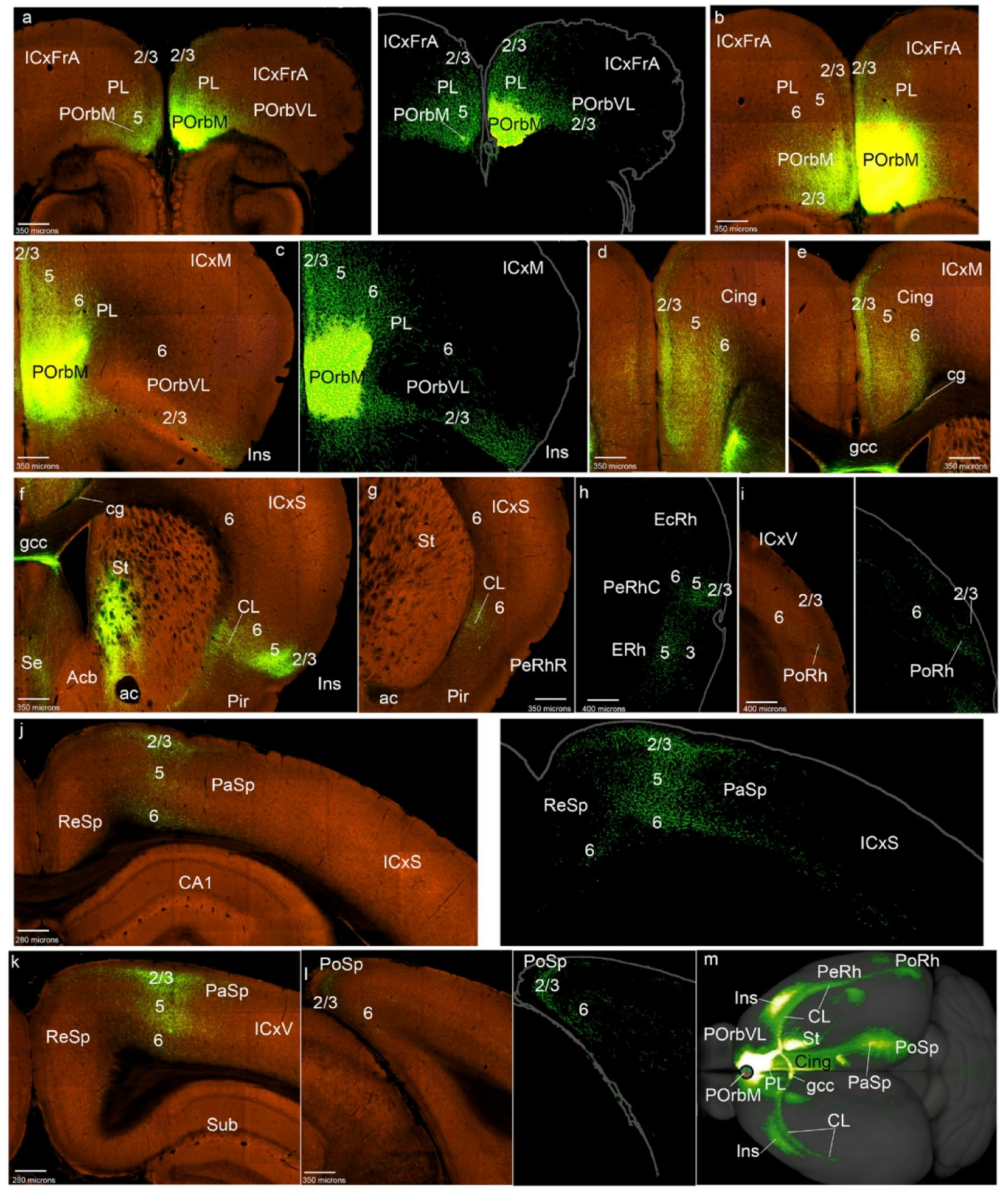
POrbM cortex projections to the mesocortical ring. **a**-**l** Analysis of the selected POrbM case shown in Fig. 1b, illustrated by means of selected coronal sections ranged from rostral to caudal, obtained from the Allen Brain database (https://brain-map.org/our-research/connectivity). Scale bars were indicated in every image. **m** Projection density map of the case analyzed in dorsal view

We describe next a case considered representative of the POrbM category, namely ORBm-0.350 (POrbM-0.350 in Figs. 1b and 2); the other 5 relevant cases of this group (Table 1) showed similar labeling (S1a). The first thing we noted was that an POrbM injection strongly labels anterogradely the contralateral POrbM, predominantly its layer 2/3, but also the infragranular layers (Figs. 2a, b). We also observed ipsilateral anterograde labeling in the adjacent ventrolateral orbitary cortex (POrbVL), mainly in the supragranular layers of this cortex; these fibers ingress into POrbVL through its layer 6 (Fig. 2a, c). The ipsilateral prelimbic cortex (PL) also appeared labeled in all its layers, except for layer 1 (Figs. 2a-c). The cingulate cortex (Cing) showed multi-layered labeling similar to that of PL (Fig. 2d, e). Furthermore, both the Cing and PL are labeled contralaterally with a similar pattern (Figs. 2a, b, d, e). At these levels we observed labeled fibers within the cingulate fasciculus, as well as fibers that cross the corpus callosum to reach the contralateral hemisphere, passing specifically through the callosal genu (cg, gcc; Figs. 2e, f).

The insular cortex (Ins) likewise appeared labeled in all its layers and the underlying claustrum was also labeled, ipsi- and contralaterally, with labeled fibers passing from the white matter to the insula through the claustrum (Fig. 2f). However, at the transition of Ins into the rostral perirhinal region (PeRhR; i.e., at coronal section levels through the crossing of the anterior commissure), the anterograde labeling was concentrated mainly in the local smaller claustral component, also ipsi- and contralaterally (Fig. 2g; see the contralateral CL in S1a). The ipsilateral caudal PeRh (PeRhC) received projections from POrbM in all its layers excepting layer 1 (Fig. 2h). Contralaterally, a similar projection was much weaker (not shown). The ectorhinal cortex is barely labeled ipsilaterally in its infragranular layers (EcRh, Fig. 2h). The PoRh also received POrbM terminals in its superficial and deep layers, mainly ipsilaterally (Fig. 2i; S1a).

The parasplenial (PaSp) cortex was also marked in all its layers, with higher-intensity ipsilateral signal in layer 2/3, and with a weak contralateral signal (Figs. 2j, k; S1a). The postsplenial cortex (PoSp) showed similar labeling as the PaSp (Fig. 2l).

On the whole, these results indicate that the POrbM projects throughout the mesocortical ring, largely bilaterally, though with ipsilateral predominance (Fig. 2m).

In relation to projections from POrbM to the isocortex (field concentric to the MCx ring), we observed slight ipsi- and contralateral labeling of the associative frontal cortex (ICxFrA; Figs. 2a, b). There was also sparse labeling in deep layers of the motor and somatosensory cortex ipsi- and contralaterally, as well as passing fibers in the white matter (for example Figs. 2e, j). The amount of labeled terminals increased in the caudal isocortex, particularly in visual areas, both in deep and superficial layers (ICxV; Fig. 2k). The portion of parahippocampal allocortex that received projections from POrbm was mainly the entorhinal area (ERh), bilaterally, mainly in its layer 5 (Fig. 2h; data not shown). The retrosplenial cortex (ReSp), which we previously reclassified as a parahippocampal area (Puelles et al. 2024), also showed terminal labeling in its deep layers 5 and 6, with scarce fibers continuing into the subiculum (Sub) and the CA fields (Figs. 2j, k). The piriform cortex was also labeled in these experiments, mainly in its deep layers (Figs. 2f, g).

Other telencephalic regions to which the POrbM projects were the medial (periventricular) striatal regions, including medial regions of the paraseptal nucleus accumbens and the olfactory tuberculum (St; Fig. 2f; TO not shown). This labeling was found also contralaterally. The septum (Se) also receives POrbM projections, both ipsi- and contralaterally (Fig. 2f).

**Fig. 3:**
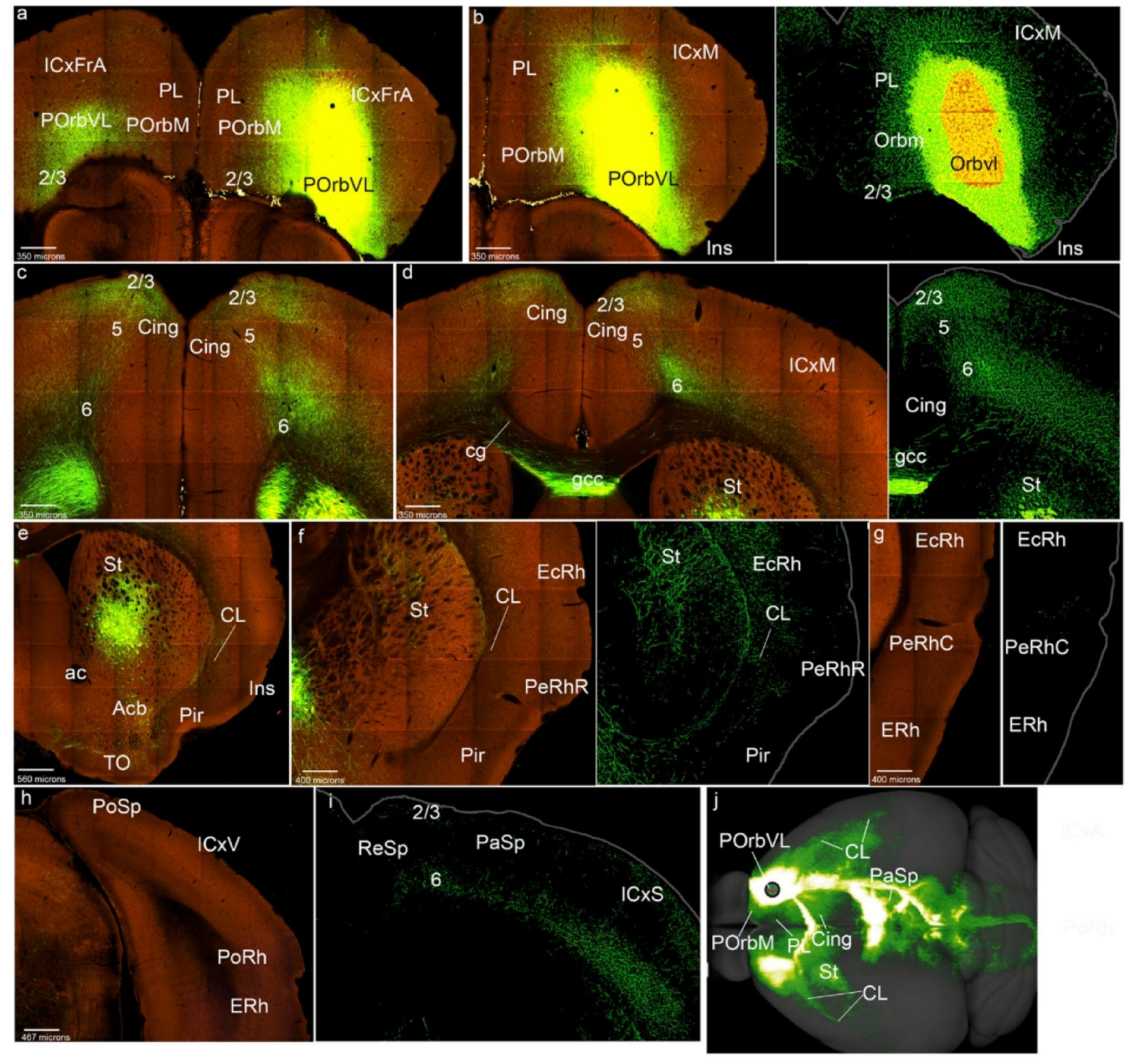
POrbVL cortex projections to the mesocortical ring. **a**-**i**) Analysis of the POrbVL case of Fig. 1c, in coronal sections ranging from rostral to caudal, obtained from the Allen Brain database (https://brain-map.org/our-research/connectivity). Scale bars were indicated in every image. **j** Projection density map of the case analyzed in dorsal view

As regards the connections of POrbvVL, we checked the cases classified as ‘ORBvl’ and ‘ORBl’ in the connectivity experiments of the Allen Brain Atlas. There were 9 cases with injection volume between 0,200 and 0.500 mm3. We describe here the case ORBl-0.317 as representative of the injections in these areas; the other cases showed similar labeling (POrbVL-o.317 in Figs. 1c and 3; S1b).

The POrbVL projected to the neighboring POrbM, mainly reaching its superficial layers 2/3, ipsi- and contralaterally (Figs. 3a, b). In contrast, the PL cortex received few POrbVL projections (in its superficial part), while the Cing cortex showed distinct labeling in all its layers except layer 1 (Figs. 3a-d). We also observed POrbM, PL and Cing labeling contralaterally. The Ins cortex received POrbVL projections mainly in its deep layers and the underlying claustrum, ipsi- and contralaterally, as occurs similarly in rostral PeRh (Figs. 3e, f). Caudal PeRh, EcRh and PoRh cortices were barely labeled in this experiment (Figs. 3g, h); however, such labeling was observed in the cases ORBvl-0.423 and ORBvl-0.296. The PoSp area was scarcely marked (Fig. 3h). The PaSp area showed labeling in layer 6 (Fig. 3i). In summary, the POrbVL projects mainly to the rostromedial side of the mesocortical ring (Fig. 3j), to the claustrum and variable projections to the rest of the ring.

As regards projections from POrbVL to isocortex, we observed connections to deep layers in associative frontal, motor, somatosensory and visual areas (ICxFrA; ICxM; ICxS; ICxV; Fig. 3a, b, d, i, j). In the allocortical ring, the retrosplenial and piriform receives cortices receive the main input from the POrbVL (ReSP; Pir; Fig. 3f, j). Other telencephalic regions to which the POrbVL projects were the medial striatal region, including the medial region of the nucleus accumbens and the olfactory tuberculum (St; Acb; TO; Fig. 3d, f, g, ). This labeling was also found contralaterally. We observed labelled fibers in the genu of the corpus callosum and in the cingulate fasciculus (gcc; cg; Fig. 3d).

### Projections from the prelimbic area to regions of the mesocortical ring and other telencephalic areas

Next, we analyzed the cases classified as ‘medial prelimbic cortex’ (PL) in the anterograde connectivity experiments of the ABMCA, also after considering their consistency with our MCx map (Puelles et al. 2024). There were 7 nominal cases with anterograde virus injection in PL and a volume between 0.200 and 0.500 mm^3^; we also added the case nominally classified as ORBm-0.208, which we reclassified as PL-0.208, as well as the similarly reclassified cases ILA-0.484 and ILA-0.205, evaluated as PL injections according to our criteria; finally, the injection in case PL-0.211 was located mainly in the cingulate cortex and passed to the Cing list. The final number of useful cases in PL was thus 9 (Table 1).

**Fig. 4:**
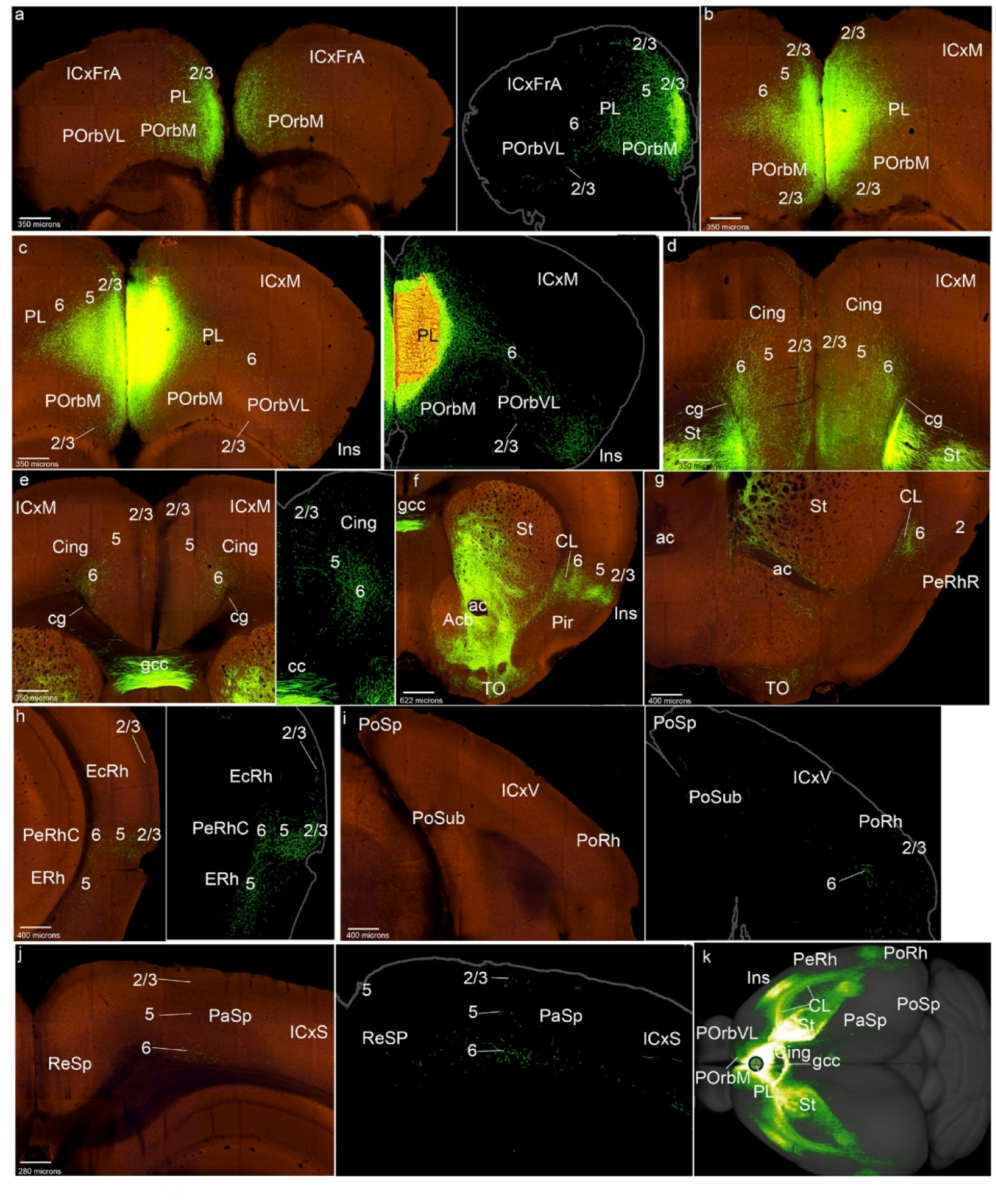
PL cortex projections to the mesocortical ring. **a**-**j** Analysis of the PL case of Fig. 1d, in coronal sections from rostral to caudal, obtained from the Allen Brain database (https://brain-map.org/our-research/connectivity). Scale bars were indicated in every image. **k** Projection density map of the case analyzed in dorsal view

We selected as the representative case PL-0.424, which clearly appears centered in the PL (PL-0.424 in Figs. 1d and 4; S2); we verified that the remaining 8 cases present similar labeling. All cortical layers of the contralateral PL excepting layer 1 were labeled. We observed projections of PL to the posterior orbitary cortices (POrbM, POrbVL), ipsi- and contralaterally, and inciding mainly on their layers 2/3, particularly rich over POrbM, and scarcely distributed in POrbVL (Figs. 4a-c). Labeled fibers pass through the layer 6 of these cortices into lateral regions of the mesocortical ring (Figs. 4a-c). All cortical layers excepting layer 1 were also labelled in the Cing areas (Figs. 4d-e). Interestingly, layer 1 was always negative in these POrb, PL and Cing experiments (Figs. 4a-e). We observed labelled fibers in the cingulate fasciculus, and also a significant number of marked fibers that pass through the corpus callosum via the genu to reach the contralateral side (cg; gcc; Fig. 4d-f). The anterior commissure also appears to be used by some of these projections to reach ipsilaterally the lateral side of the ring (i.e., the claustrum in the insular region) (Fig. 4g).

The Ins region also appeared labeled bilaterally in all its layers, together with negative patches intermixed with labeled ones in the claustrum (Fig. 4f). In the rostral perirhinal cortex, labeling in the external layers was weak, contrasting with the patchy terminals in the claustrum, also contralaterally (PeRhR; Fig. 4g). The caudal PeRh appeared labeled in all its layers, also contralaterally (PeRhC; Fig. 4h). In contrast, the EcRh only showed PL terminals in its superficial and deep layers, similarly as the PoRh area (Figs. 4h, i). The parasplenial (PaSp) and postsplenial (PoSp) ring sectors appear weakly labeled, especially the latter; these projections are located mainly in their deep layers (Figs. 4h, i). In summary (Fig. 4k), PL projects mainly to adjacent POrb and Cing areas, and labels somewhat differentially the lateral side of the ring (Ins, PeRh, EcRh, PoRh).

As regards projections to isocortical regions, the PL data are similar to those in POrbM injections: PL projects sparsely to the associative frontal cortex, mainly targetting superficial layers (ICxFrA; Fig. 4a). Deep layers in motor, somatosensory, and visual regions also showed some marked PL fibers (layer 6, ICxM, ICxS, ICxV; Figs. 4b-e, i, j). In the allocortical ring the entorhinal cortex and the piriform cortex are the main domains receiving PL inputs (ERh; Pir; Figs. 4f, h).

With respect to other telencephalic regions, the PL cortex projects to regions of the medial striatum, including the accumbens and olfactory tuberculum (St, Acb, TO; Figs. 4f, g). The septum also receives PL inputs (Fig. 4f).

### Projections from the cingulate area to regions of the mesocortical ring and other telencephalic areas

To analyze the cingular cortex (Cing), we first checked the cases classified in the anterograde connectivity experiments of the Allen Brain Atlas as dorsal or ventral anterior cingulate area (ACAd, ACAv). The number of cases with anterograde injection in ACAd and a volume between 0.200 and 0.500 mm3 was 8. The number of cases with anterograde injection in ACAv and a volume between 0.200 and 0.500 mm3 was 4. We did not find any nominally retrosplenial cases that might be reclassified into the posterior cingulate area. We added to our Cing list the nominal PL-0.211 case mentioned above that was reclassified as Cing-0.211, thus reaching a total of 11 useful cases.

**Fig. 5:**
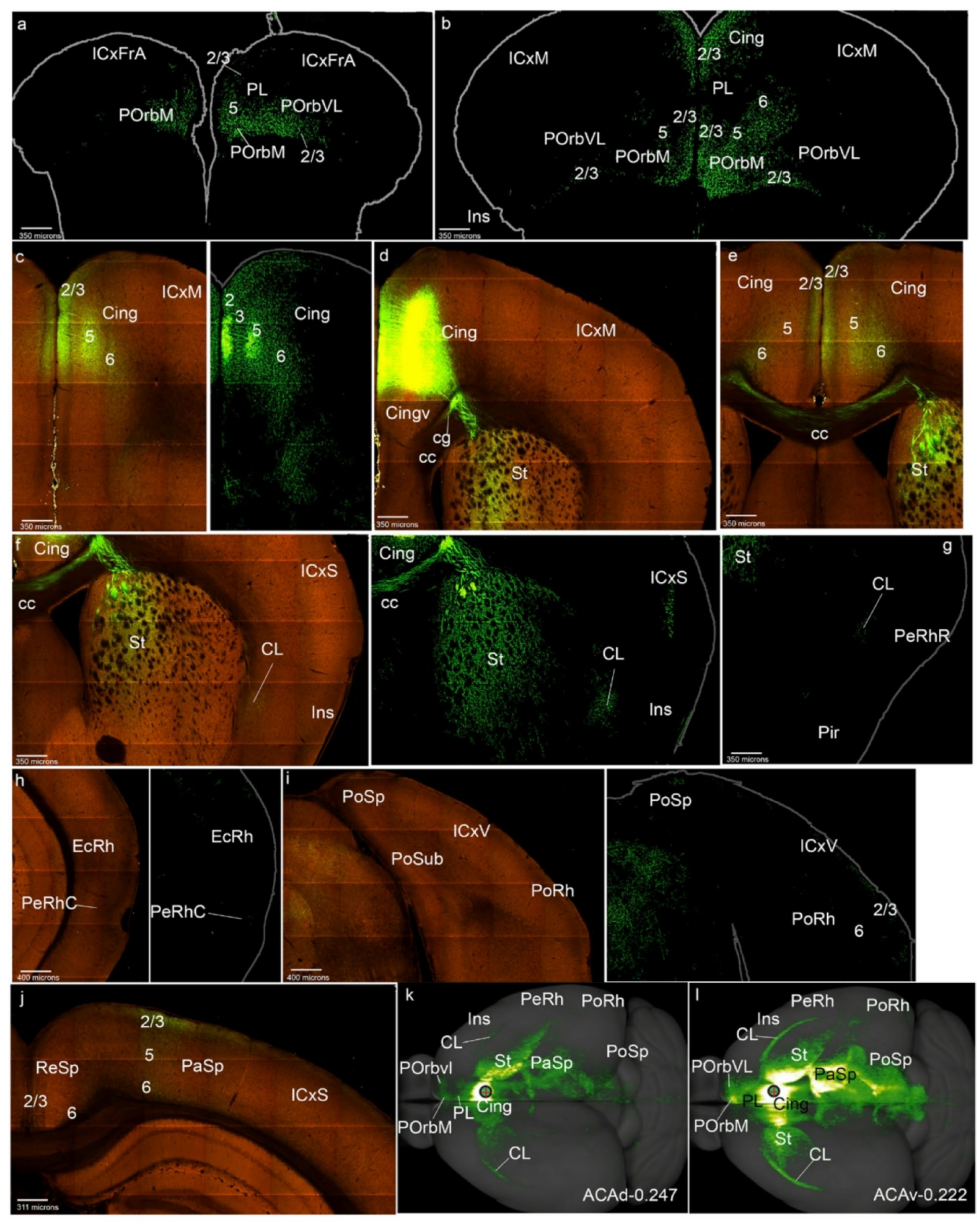
Cing cortex projections to the mesocortical ring. **a**-**j** Analysis of the Cing case of Fig. 1e, in coronal sections from rostral to caudal, obtained from the Allen Brain database (https://brain-map.org/our-research/connectivity). Scale bars were indicated in every image. **k**, **l** Projection density maps of the case analyzed and an additional case mentioned in the text -ACAv 0.222- in dorsal view

As representative for description, we selected ACAd-0.247 (Cing-0.247 in Figs. 1e and 5; S3) and compared with the other cases. The Cing cortex rostrally and caudally to the injection appeared bilaterally labeled in all layers (except layer 1; Figs. 5b-e). Distinct labeling appears in layers 2/3 and 5 in POrbM, and layer 2/3 in POrbVL, covering bilaterally these two areas, as well as the underlying posterior orbitary layer 6 (Figs. 5a, b). The intercalated PL area instead received cingulate afferents in layer 2/3 and more abundant ones in its layer 6, both ipsi- and contralaterally, though part of this labeling might be fibers passing towards POrb and lateral parts of the ring (Figs. 5a, b). The Ins and rostral PeRh cortices received bilaterally Cing inputs concentrated mainly in the respective claustrum components (Ins; PeRhr; Fig. 5f, g). The caudal PeRh showed scarce Cing projections, and PoRh displayed labeling in its layers 2/3 (PeRhC; PoRh; Figs. 5h, i). The PaSp showed bilateral labeling in its deep and superficial layers (Fig. 5j), whereas PoSp barely receives terminals (Fig. 5i). Labeled fibers passed to the contralateral side through the corpus callosum (cc; Fig. 5e, f). To project within the medial side of the mesocortical ring, the Cing efferents used the cingulate fasciculus (cg; Fig. 5d). The projection density map of the case selected as representative (ACAd-0.247) shows little contralateral claustrum labeling, whereas a similar case ACAv-0.222 distinctly shows claustrum labeling (CL; Figs. 5k, l).

Concentrically to the mesocortical ring the Cing also projects sparsely into the layer 2 of ICxFrA region (Fig. 5a) as well as into deep and superficial layers of the somesthetic and visual cortices (ICxS; ICxV; Fig. 5f, i, j). The main labeling reaching the allocortex was observed in the restrosplenial area (Fig. 5j).

With respect to other labeled telencephalic regions, we found that the striatum is labeled medially (Figs. 5d-f). Contralateral fibers pass through the corpus callosum (Figs. 5d-f).

Regarding the cases of the ventral cingulate region, according to the Allen Brain Atlas classification, these cases showed similar projections as the ACAd (we show the density projection map of ACAv-0.222 in Figs. 5l and S3).

### Projections from the parasplenial and postsplenial areas to regions of the mesocortical ring and other telencephalic areas

Recently, Puelles et al. (2024) coined the terms *parasplenial* (PaSp) and *postsplenial* (PoSp) for caudomedial mesocortical areas located adjacent to the retrosplenial cortex (PaSp) or just caudal to it (PoSp; this is a peculiarly thin caudomedial small transitional area between PaSp and PoRh). In the connectivity experiments of the Allen Brain Atlas, this terminology and classification is not yet recognized. Therefore, to find injections in these regions, we looked for candidate PaSp/PoSp injections among the nominally ‘retrosplenial’ and ‘medial/posterior associative visual’ injections, using the three-dimensional Allen injection map. We checked in coronal sections the positions of the candidate injections relative to our map of the local mesocortical ring and also examined whether the connections elicited by these experiments were significantly comparable to those of the other mesocortical ring sectors. As in the previous cases, we only considered experiments with an injection volume between 0.200 and 0.500 mm^3^.

**Fig. 6:**
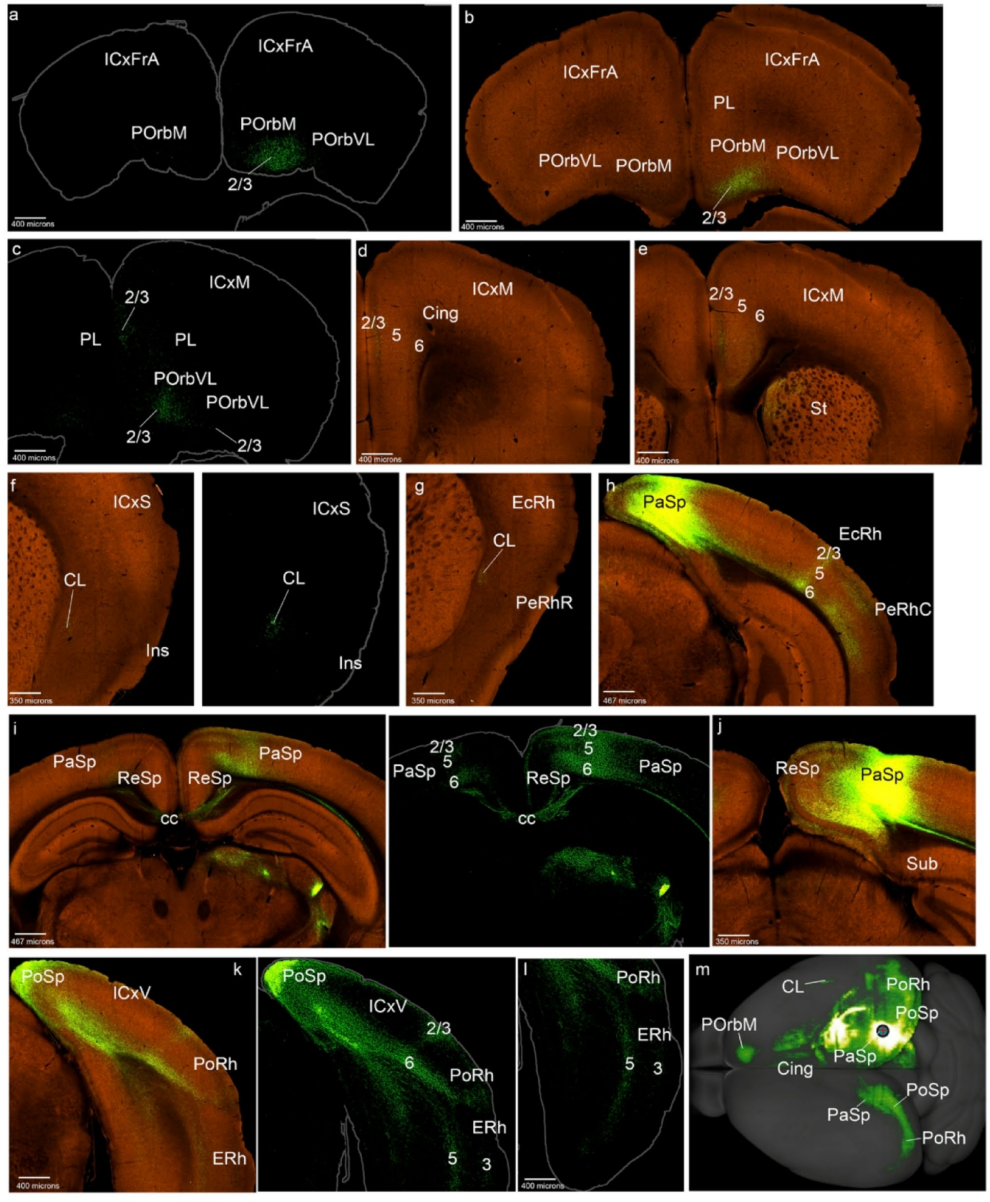
PaSp cortex projections to the mesocortical ring. **a**-**l** Analysis of the PaSp case of Fig. 1f, in coronal sections from rostral to caudal, obtained from the Allen Brain database (https://brain-map.org/our-research/connectivity). Scale bars were indicated in every image. **m** Projection density map of the case analyzed in dorsal view

Various cases categorized in the Allen connectivity map as located at the *retrosplenial area*,* lateral agranular part* (RSPagl) and *posteromedial visual area* (VISpm) correspond topographically to PaSp. We found 6 cases in these areas with a convenient injection volume (Table 1). We analyzed as representative the case VISpm-0.236 (PaSp-0.236 in Figs. 1f and 6; S4a) and compared it with other cases labeled in the same area. The injection in the PaSp cortex labeled the ipsilateral POrbM and PL areas, both superficial and deep layers (Figs. 6a-c), with weaker labeling in the contralateral side. The labeling extended likewise to POrbVL (Figs. 6a-c). The Cing cortex also displayed superficial and deep layer labeling (Figs. 6d, e). We observed scarce labeling in the contralateral side of all these areas (Fig. 6a, b, c, e).

We detected very weak bilateral labeling in the insular claustral nucleus but no labeling in insular cortical layers (Fig. 6f). The rostral PeRh showed similar labeling as the Ins (Fig. 6g). Caudal PeRh and EcRh cortices appeared labeled in their superficial and deep layers (Fig. 6h). The ipsilateral PaSp lying rostral and caudal to the virus injection was labeled in all cortical layers bilaterally (Figs. 6h-j). PoSp also showed similar anterograde labeling as the rostral PaSp (Fig. 6k). The PoRh distinctly received PaSp terminals in its deep and superficial layers (Fig. 6k). Contralateral projections in all these regions mentioned were scarce except for PaSp. The contralateral side was reached through a caudal region in the corpus callosum (Fig. 6i). In summary, PaSp projects mainly ipsilaterally to most caudal and medial regions in the mesocortical ring, including the POrbM (Fig. 6m).

Concentrically to the mesocortical ring, PaSp projects mainly to superficial and deep layers of the visual cortex (ICxV; Fig. 6k). As regards the allocortex ring we observed PaSp inputs in the retrosplenial and entorhinal cortices (Figs. 6i-l). PaSp also projects to the medial periventricular region of the caudate nucleus (Fig. 6e) and to the ipsilateral medial septum (Fig. 6e).

**Fig. 7:**
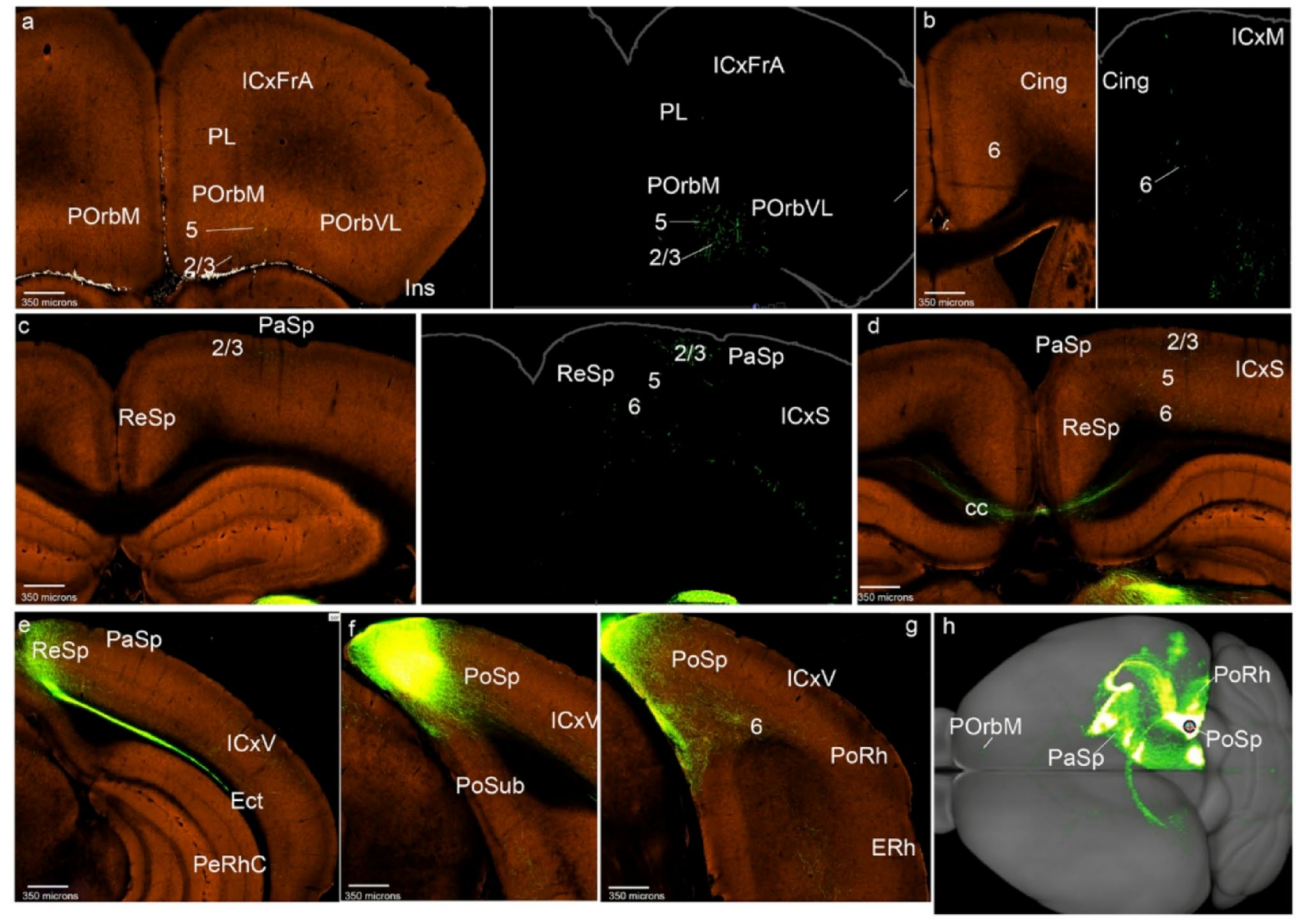
PoSp cortex projections to the mesocortical ring. **a**-**g** Analysis of the PoSp case of Fig. 1g, in coronal sections from rostral to caudal, obtained from the Allen Brain database (https://brain-map.org/our-research/connectivity). Scale bars were indicated in every image. **h** Projection density map of the case analyzed in dorsal view

We also analyze next the case RSPagl-0.105 as representative of the postulated mesocortical PoSp area (PoSp-0.105 in Figs. 1g and 7; S4b). Cases at this locus were scarce; after examining the coronal sections, we chose this case that has a smaller injection volume than that we normally accept. We observed distinct PoSp inputs in layers 2/3 and 5 of POrbM, which barely extended to POrbVL (Fig. 7a). The Cing and PL cortices were sparsely labeled in their layer 6, possibly representing fibers passing on their way to POrbM (Figs. 7a, b). These inputs were found only ipsilateral to the injection. Within the PaSp area, the terminals coming from the PoSp decreased caudorostrally, with contralateral fibers arriving mainly at caudal PoSp/PaSp levels (Figs. 7c-e). The PoSp injection labeled the contralateral PoSp area in its deep and superficial layers (Figs. 7f, g). The Ins and PeRh cortex were not labelled. Finally, the PoRh also received PoSp inputs (Fig. 7g). Similar to PaSp, PoSp projects mainly to the caudomedial part of the mesocortical ring, but also showed significant labeling in POrbM (Fig. 7h; note labelling in POrbM is not visible in this density map).

Concentrically to the mesocortical ring we found PoSp inputs in the visual isocortex (Fig. 7e) and the entorhinal allocortex (ICxV; ERh; Fig. 7e-g). The striatum was sparsely labeled in the periventricular caudate nucleus (not shown). The fibers passing to the contralateral side cross in the caudalmost splenial region of the corpus callosum (Fig. 7d).

### Projections from the postrhinal cortex to regions of the mesocortical ring and other telencephalic areas

**Fig. 8:**
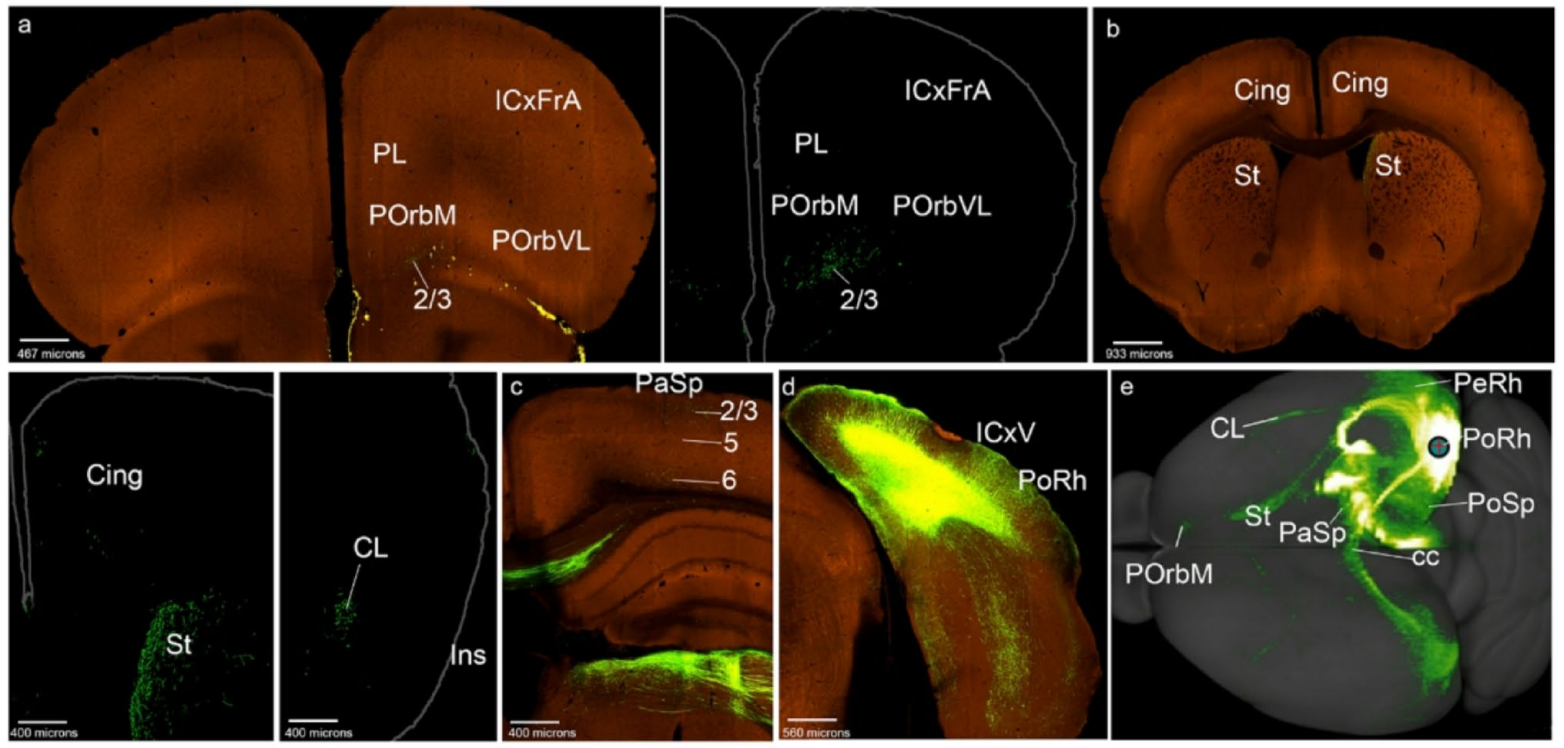
PoRh cortex projections to the mesocortical ring. **a**-**d**) Analysis of the PoRh case of Fig. 1h, in coronal sections from rostral to caudal, obtained from the Allen Brain database (https://brain-map.org/our-research/connectivity). Scale bars for both cases were indicated in every image. **e** Projection density map of the case analyzed, in dorsal view

The case chosen as representative for injections in the PoRh region was VISpor-0.319 (reclassified as PoRh-0.319); we only disposed of two cases credibly injected in this area that have the appropriate virus injection volume (Table 1; Figs. 1h; S5a). The projections observed were similar to the pattern which is described below for the EcRh cortex. Since we have a larger number of cases marked in EcRh (5 cases), this type of labeling will be explained better in the corresponding section. In summary, The PoRh cortex projects to the superficial layers of POrbM (Fig. 8a). It scarcely connects with the PL and Cing cortices (Fig. 8a, b). It does label the claustrum at the Ins level and PeRh (Fig. 8b). The PaSp cortex, and other caudal regions of the mesocortical ring outside the injection also receive PoRh inputs (Fig. 8c-e). Outside the ring, we observed PoRh labeling in deep and/or superficial layers of the visual isocortex (Fig. 8d). The striatum receives PoRh inputs medially (Fig. 8b).

### Projections from the perirhinal cortex to regions of the mesocortical ring and other telencephalic areas

To analyze injections in (agranular) PeRh and (dysgranular/granular) EcRh cortices we studied the injections catalogued as ‘VISC, AI and VISpor’ of the Allen Brain experiments with a volume between 0.200 and 0.500 mm^3^ (Table 1).

**Fig. 9:**
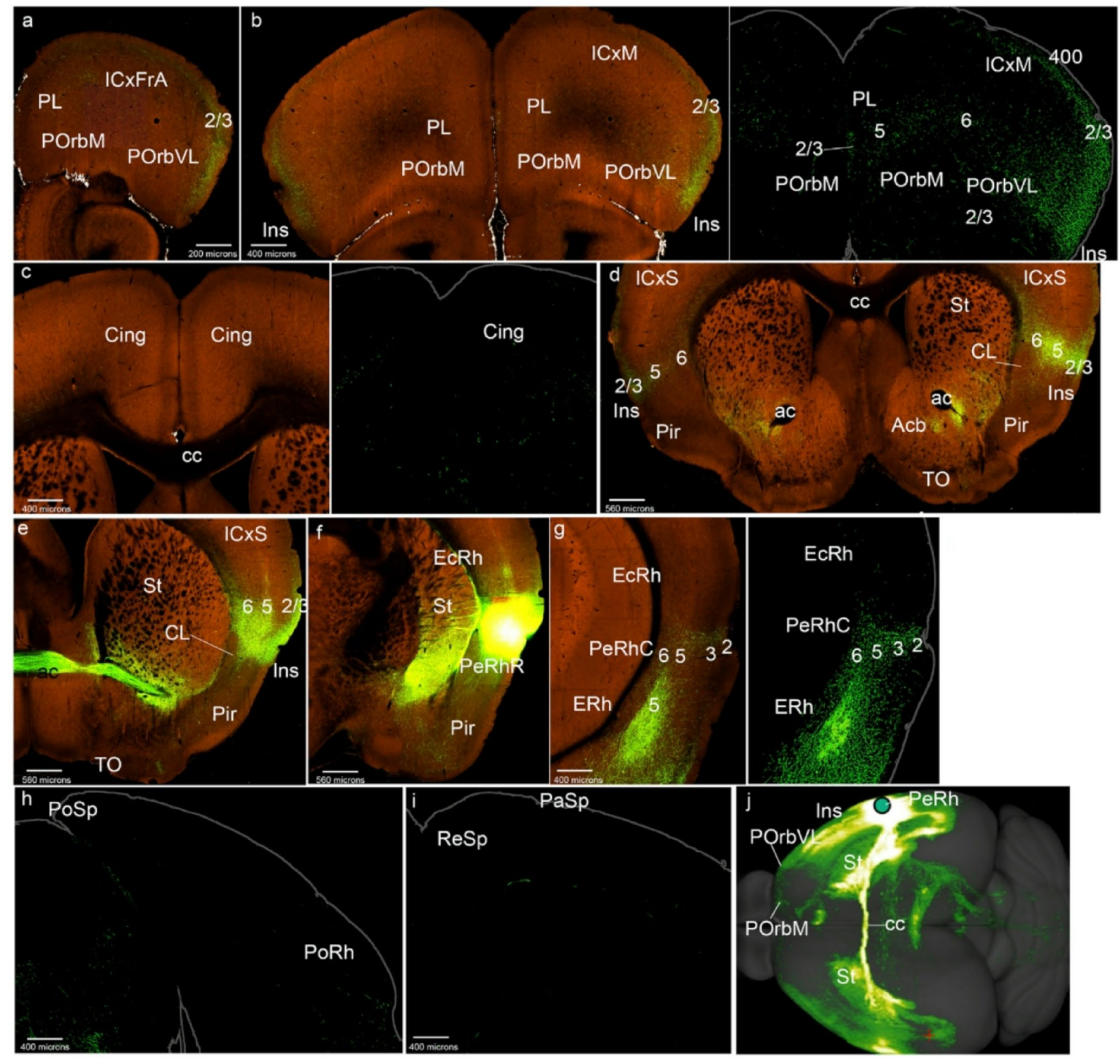
PeRh cortex projections to the mesocortical ring. **a**-**i** Analysis of the PeRh case of Fig. 1i, in coronal sections from rostral to caudal, obtained from the Allen Brain database (https://brain-map.org/our-research/connectivity). Scale bars were indicated in every image. **j** Projection density map of the case analyzed in dorsal view

We obtained 5 cases injected in PeRh (Table 1). To describe the representative PeRh projections, we examined the case VISC-0.450 (PeRh in Figs. 1i and 9; S5b). The POrbM area received bilateral inputs from the PeRh cortex, mainly in its superficial layers 2/3 (Figs. 9a, b). The POrbVL showed superficial and deep inputs (Fig. 9b). The PL displayed PeRh inputs in its deep and superficial layers (Figs. 9a, b). The Cing shows scarce anterograde labelling (Fig. 9c; but see case VISC-0.448). The Ins cortex was labeled in all layers except layer 1, also in the contralateral side, with less abundant terminals in the claustrum (Figs. 9d, e). Parts of the PeRh cortex distant from the injection site showed ipsilateral and contralateral inputs in superficial and deep layers, similar to Ins (Fig. 9f, g). The PoRh also showed PeRh inputs in superficial and deep layers (Fig. 9h, this result is clearer in the VISC 0.488 case). The PaSp and PoSp areas did not receive PeRh inputs (Figs. 9h, i). In summary, PeRh sends efferents mainly to the lateral and rostral parts of the mesocortical ring (Fig. 9j).

Various isocortical areas were labeled by PeRh projections at different rostrocaudal levels in deep and superficial layers (ICxFrA; ICxM; ICxS; Figs. 9a, b, d, e). The ERh domain in the allocortex ring showed PeRh inputs in superficial and deep layers (Fig. 9g). In the same ring, the Pir cortex also showed PeRh inputs (Figs. 9d-f). Other telencephalic projections were observed, at the lateral striatum, extending into the accumbens nucleus and the olfactory tuberculum (St; Acb; TO; Figs. 9d-f). Both the corpus callosum and the anterior commissure carry labelled fibers to the contralateral hemisphere (cc; ac; Figs. 9e, j).

There is another relevant case with a caudal PeRh injection (Table 1, S5, VISpor-0.355). In this case the PeRh projections were similar to PoRh ones.

**Fig. 10:**
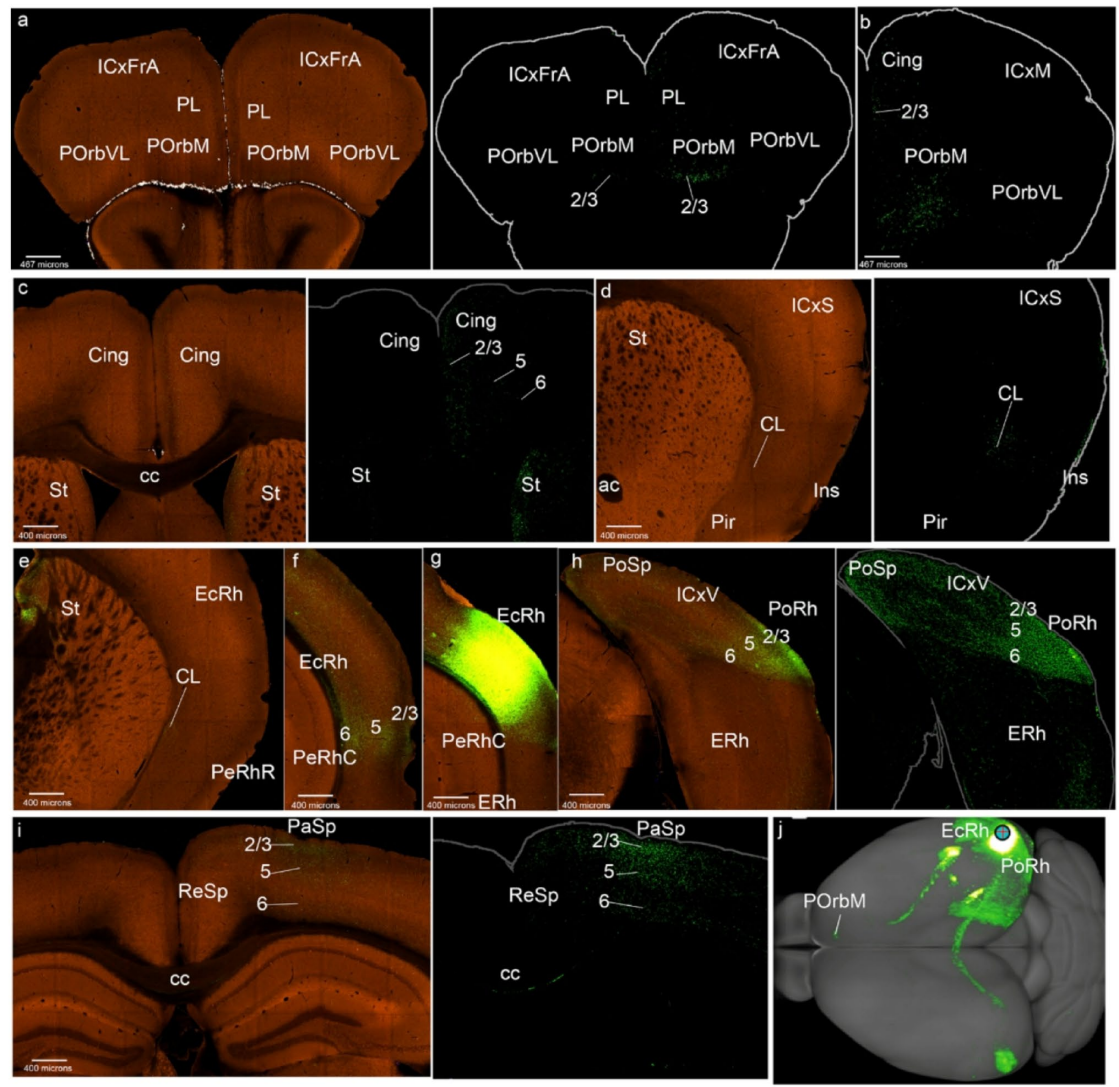
EcRh cortex projections to the mesocortical ring. **a**-**i** Analysis of the EcRh case of Fig. 1j, in coronal sections from rostral to caudal, obtained from the Allen Brain database (https://brain-map.org/our-research/connectivity). **j** Projection density map of the case analyzed, in dorsal view

For the EcRh cortex, we observed 5 cases with injection in this mesocortical region (Table 1). The chosen representative case was VISpor-0.367 (Figs. 1j and 10; S5c). In this experiment, we observed EcRh inputs in the POrbM cortex, mainly in layer 2/3 (Fig. 10a). The PL and Cing were also labeled mainly in superficial layer 2/3 but also in deep layers (Figs. 10a-c). All these structures were labeled also contralaterally (Figs. 10a, c). The claustrum received inputs from EcRh at Ins and PeRh levels (Figs. 10d, e). The insular and posterior orbital areas were labeled more clearly in other EcRh experiments such as VISpor-0.310 and 0.360 (Fig. S5c). The PeRh and EcRh areas caudal to the injection showed labeling in all layers (except layer 1), like in PoRh (Figs. 10f-h). Finally, PoSp and PaSp received also inputs in all layers (Figs. 10h, i). In summary, EcRh projects mainly to caudal areas of the mesocortical ring, but also labeled distinctly the POrbM (Fig. 10j). As we mentioned before, the injections in PoRh produce a similar pattern of labeling (compare the density maps in S5a and c).

Outside of the mesocortical ring, EcRh sends projections to caudal isocortex (superficial and deep layers; ICxV; Fig. 10h). In the allocortex, we detected EcRh afferents in the ERh region (Fig. 10g, h). The striatum is labeled mainly in its medial area (St; 10c, e). The contralateral side was reached through the caudal corpus callosum (cc; Fig. 10i).

### Projections from the insular cortex to regions of the mesocortical ring and other telencephalic areas

**Fig. 11:**
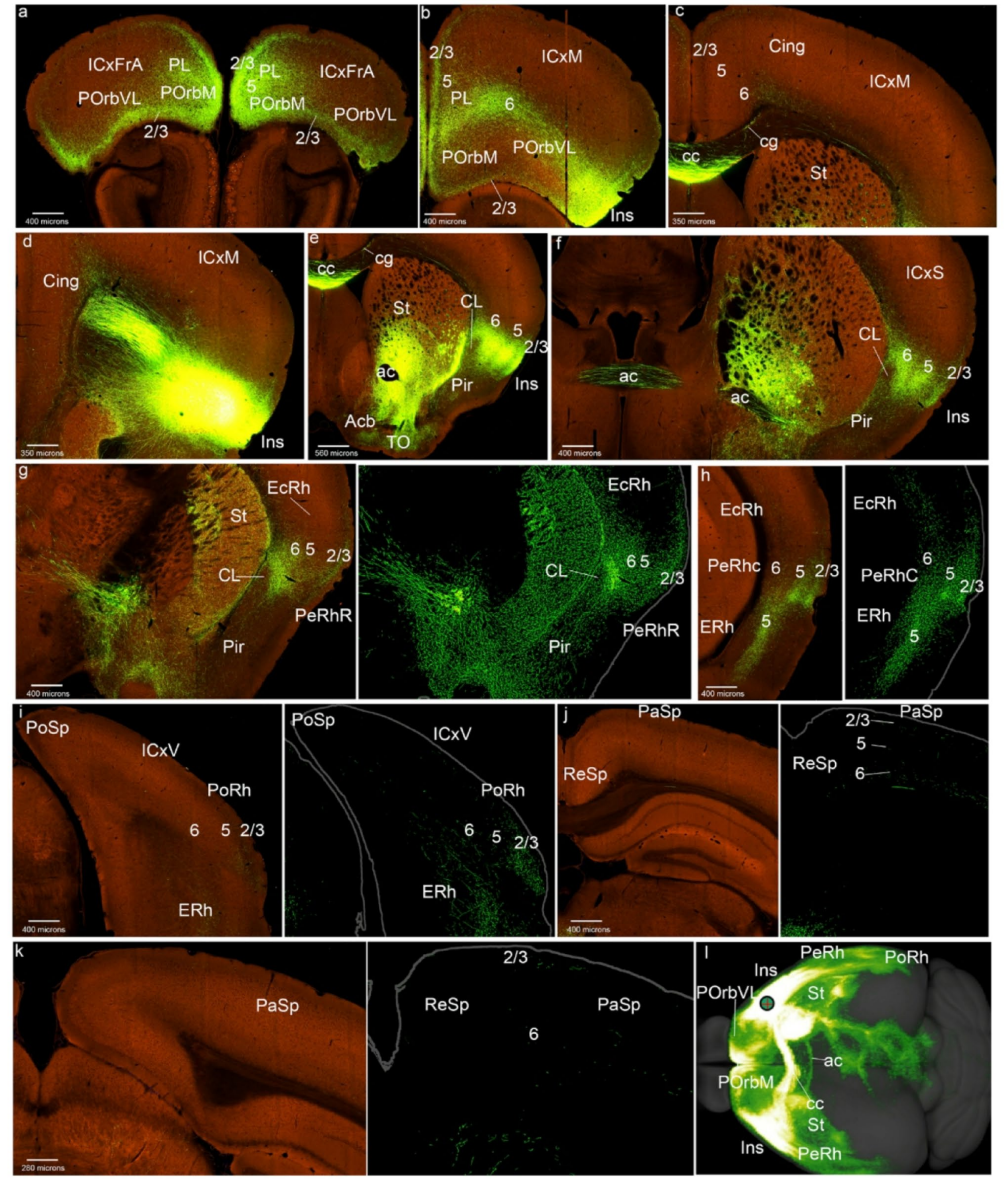
Agranular Ins cortex projections to the mesocortical ring. **a**-**k** Analysis of the agranular Ins case of Fig. 1k, in coronal sections from rostral to caudal, obtained from the Allen Brain database (https://brain-map.org/our-research/connectivity). Scale bars were indicated in every image. **l** Projection density map of the case analyzed in dorsal view

**Fig. 12:**
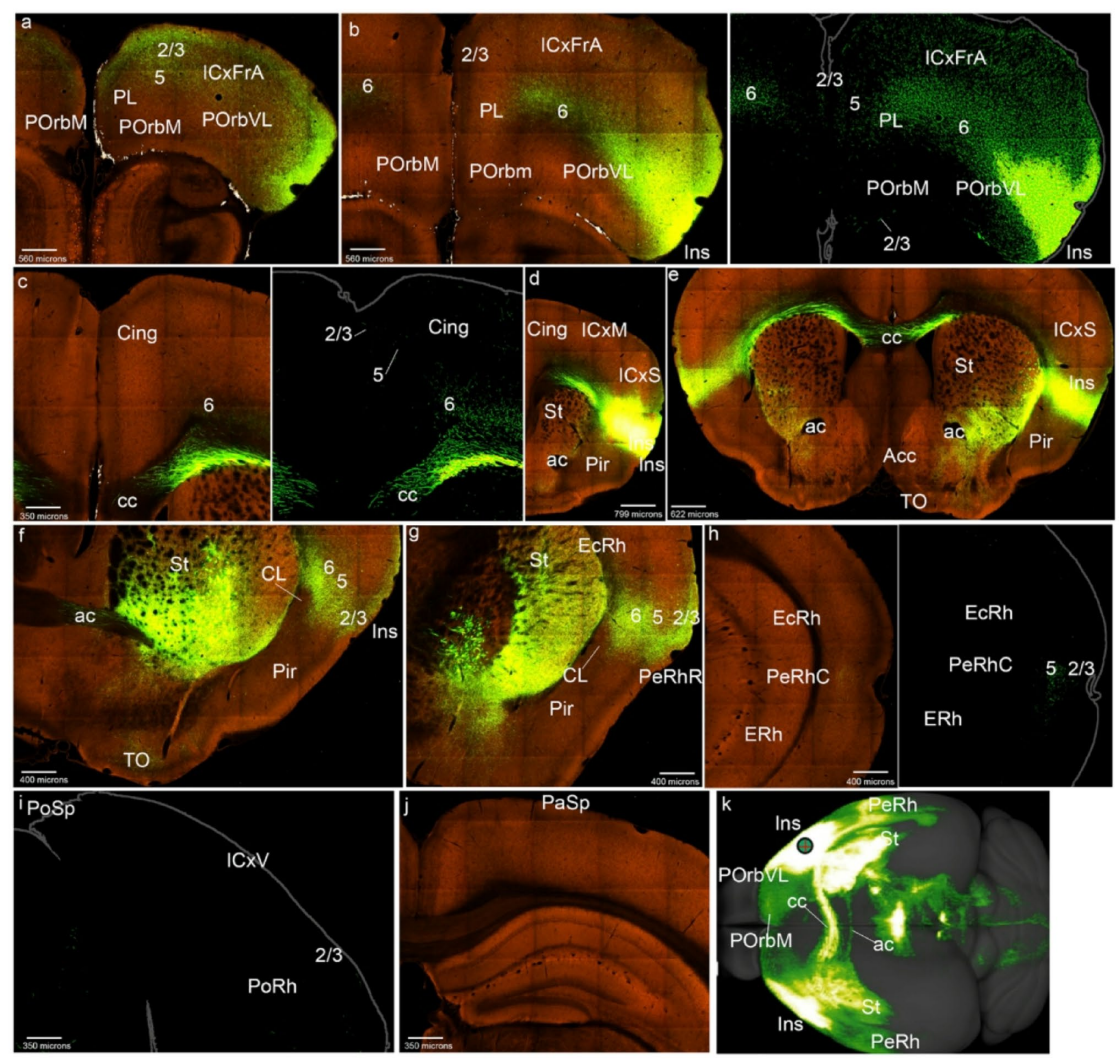
Dysgranular/granular Ins cortex projections to the mesocortical ring. **a**-**j** Analysis of the dysgranular/granular Ins case of Fig. 1l, in coronal sections from rostral to caudal, obtained from the Allen Brain database (https://brain-map.org/our-research/connectivity). Scale bars were indicated in every image. **k** Projection density map of the case analyzed in dorsal view

We examined separately experiments labeling the agranular, dysgranular and granular insular cortex. There were initially 9 injections with an injection volume between 0.200 and 0.500 mm3 marked as located within the anterior insula area (AI) in the connectivity experiments of the Allen Brain Atlas, but we reclassified two of them (cases AIp-0.280 and AIp-0.245) as lying rather at the olfactory or perirhinal cortices, respectively. The total of useful insular cases was thus 7: cases AId-0.212 and AIv-0.416 were injected in the *agranular* insular cortex; cases AId-0.398, AId-0.473 and AId-0.243 were injected in the *dysgranular*/*granular* insular cortex; cases AIv-0.355 and AIv-0.349 were injected in the claustrum. We selected for description a case representative of granular insular cortex, AId-0.416 (Figs. 1g and 11 and S6a), and a case representative of dysgranular/granular cortex, AI-0.398 (Figs. 1h and 12 and S6b). Data from claustrum injections appear in Supplementary material (S6c).

We analyzed case AIv-0.416 as representative of the injections in the agranular insular cortex and compared it with similar injections (Ins-0.416 in Figs. 1k and 11; S6a). The POrbM and POrbVL mesocortical areas appeared labeled by insular inputs in their superficial layers 2/3 and deep layers 5 and 6, ipsi- and contralaterally (Figs. 11a, b). The PL and Cing cortices showed similar labeling in their superficial and deep layers (Figs. 11a-d). Layer 6 labeling may represent passing fibers (Fig. 11b). Caudally to the Ins injection, all the layers in the caudal Ins cortex, plus the underlying claustrum were labeled ipsi- and contralaterally (Figs. 11d-f). The PeRh cortex showed similar labeling as the insula in its rostral part (Fig. 11g). The claustrum component in both areas showed less Ins inputs that the cortical layers (Figs. 11d-g). More causally, the PeRh showed all layers labeled (except layer 1; Fig. 11h). The EcRh received insular inputs mainly in its layers 2/3 and 6 (Figs. 11g, h). The PoRh cortex also was marked in all its layers (Fig. 11i). The PoSp area was not clearly marked in this material (Fig. 11i). The PaSp is weakly labeled in layer 6, and also superficially (Figs. 11j, k). All the projections described in the mesocortical ring were also observed contralaterally, with pathways to the other hemisphere through the corpus callosum and the anterior commissure (Fig. 11c, e, f). The cingulate tract is also marked within the ipsilateral ring (Fig. 11c, e). In summary, the agranular insular cortex sends efferents to the lateral and medial mesocortical ring, with less presence at caudal-medial levels (Fig. 11).

We analyzed case AId-0.398 as representative of the injections in the disgranular/granular insular cortex and compared it with similar injections (Ins-0.398 in Figs. 1l and 12; S6b). Efferent projections from the disgranular/granular insular cortex reach POrbM, in its layer 2/3, both ipsi- and contralaterally (Figs. 12a, b). The POrbVL cortex appears marked in its deep layer 6 (Figs. 12a, b). The PL cortex showed insular inputs in its superficial and deep layers, bilaterally (Figs. 12a, b). The Cing region showed labeling mainly in its deep layers (Fig. 12c).

The injections distinctly labeled the contralateral Ins area (all cortical layers; Figs. 12d, e). As regards the lateral and caudal parts of the MCx ring, the ipsi- and contralateral rostral and caudal PeRh cortex also received disgranular/granular insular projections in most cortical layers (except layer 1; Figs. 12g, h). The claustrum in both regions showed less inputs than the cortical layers (Fig. 12f-h). The EcRh area was also labeled, except in its caudal part (Fig. 12g, h). The PoRh area received very few projections from this injection (Fig. 12i). PoSp and PaSp are devoid of projections in these experiments (Figs. 12i, j). As can be seen in Fig. 12k, the projections of the disgranular/granular insula are ipsilateral and contralateral, though they are hardly present in the medio-caudal regions of the mesocortical ring.

In this material the isocortex appears labeled in deep and superficial layers at different loci (ICxFrA; ICxM; ICxS; ICxV; Figs. 11a-d, f and i and 12a, b, d and e); also the motor cortex shows a similar pattern (not shown). In the allocortical ring, the main regions labeled were the piriform and entorhinal cortices (Figs. 11e-i and 12d-h, Ins g/dys did not labelled ERh). Other regions in the telencephalic vesicle showing disgranular/granular insular projections were the lateral striatum, including the accumbens nucleus and the olfactory tuberculum (St; Acb; TO; Figs. 11c-g and 12d-g). We also observed that both the corpus callosum and the anterior commissure carry labeled fibers to the contralateral hemisphere (cc; ac; Figs. 11c-f and 12c-f).

There were cases with virus injection in the insular claustrum (Table 1 and S6c). In these cases, the main input/labeling is in the claustrum itself all along the lateral half of the mesocortical ring, but the labeling also extends into the orbital claustrum and the POrbM. There was no contralateral labeling in these cases (S6c). There was also a case with viral injection mainly in the smaller perirhinal part of the claustrum with a labeling similar to that mentioned for the insular claustrum, but displaying contralateral projections (Table 1, S5b, case AIp-0.245).

## Discussion

The aims of the present work were: (a) to investigate qualitatively the existence of a network of inter- connections within the mesocortical ring, which may prove to have functional implications. (b) to examine evidence suggesting a hierarchy within the ring, where a particular sector may organize the overall state and output of the mesocortex.

### Internal connections within the mesocortical ring

We recently redefined the limbic mesocortex within the cortical concentric-ring theory (Puelles et al. 2024). It is conceived as a specialized cortical structure intercalated all around between isocortex and allocortex that displays an unique molecular profile (83 gene markers at this date, many of them related to synaptic protein properties; Puelles et al. 2019, 2024; Puelles and García-Calero 2026 in press). Our definition is consistent topographically with previous observations on high activity of kainic receptors in layers 5 and 6 and low degree of myelination (Palomero-Gallagher and Zilles 2004, 2015). We noted likewise a disgranular layer 4 characterizing its 6-layered cytoarchitecture. All these features are absent in all parts of the allocortex, including the retrosplenial cortex, and mark salient differences with all isocortical areas (Puelles et al. 2024).

The mesocortical limbic ring is subdivided into several sectors, whose number and precise identity were recently updated using the newly discovered molecular criteria (46 selective gene markers in Puelles et al. 2024; corroborated with 37 additional markers by Puelles and García-Calero 2026 in press). The literature already amply supports the conclusion that there exist strong interconnections between some of these sectors, though the available results mainly refer selectively to the POrbM, Cing and Ins cortices, which have attracted most attention as instances of limbic emotion-related areas (e.g., Jones et al. 2005; Nagai et al. 2010; Aggleton 2012; Rolls 2019a,b; Du et al. 2020; Fermin et al. 2022). We aimed to investigate specifically potential intrinsic interconnections throughout the cortical limbic ring, and thus examined anterograde projections of all mesocortical sectors, thanks to the public availability of many relevant experiments at the ABMCA. We found that each ring sector shows some preferences as regards its targets within the MCx ring (Figs.S1-S6). Normally each of them projects abundantly ipsi- and contralaterally onto neighboring ring sectors in both directions of the ring, though less abundant projections reach nevertheless one or more distant ring sectors (even diametrically opposed ones), always including, interestingly, the POrbM area. The latter sector, which accordingly receives input from all parts of the mesocortical ring, also shows the most widespread pattern of efferences, since it reaches relatively uniformly the whole ring with its output. The sum of these diverse partial intrinsic mesocortical relays represents a complete bilateral ring-shaped pathway that is stronger ipsilaterally than contralaterally.

**Fig. 13:**
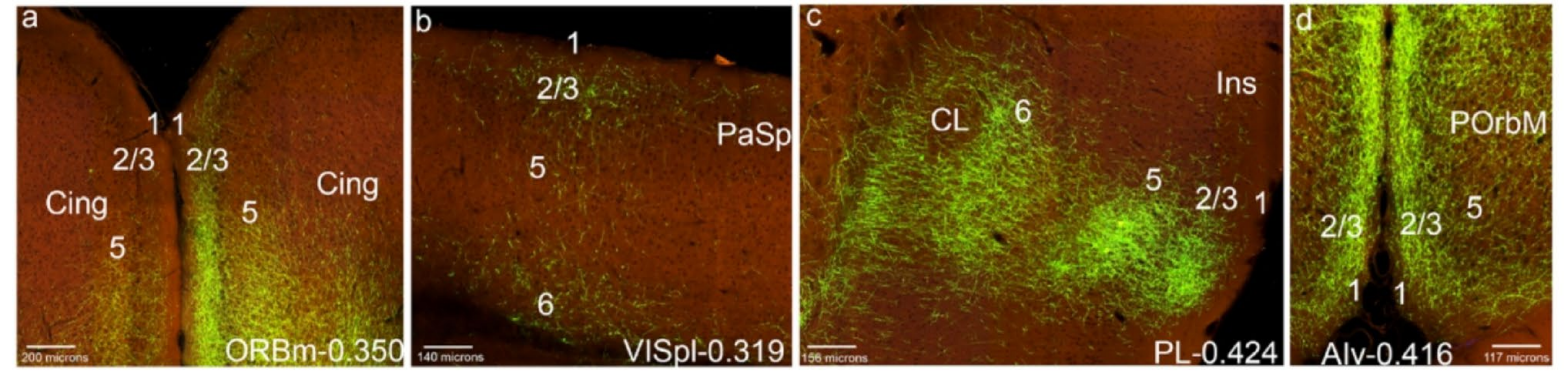
Layers labelled anterogradely in the mesocortical internal connectivity pattern. Detail views of some of the experiments analyzed showing the layers that receive projections at different locations. **a** Injection in POrbM, detail of Cing labelled layers. **b** Injection in PoRh, detail of PaSp labelled layers. **c** Injection in PL, detail of Ins labelled layers. **d** Injection in agranular Ins, detail of POrbM labelled layers

The details of synaptic connectivity relative to these intrinsic terminals are still unknown (in our material strong mesocortical intrinsic connections target usually layers 2–6 (Fig. 13), a peculiar cortico-cortical pattern not previously highlighted by general studies of cortico-cortical connections (e.g., Barbas 1986, 2015; Barbas and Rempel-Clower 1997; Barbas and Zikopoulos 2006; García-Cabezas and Barbas 2017; García-Cabezas and Zikopoulos 2019; García-Cabezas et al. 2019, 2023; Aparicio-Rodríguez and García-Cabezas et al. 2023; Zaldivar-Díaz and García-Cabezas 2026 in press). Some studies using different markers observed cortico-cortical projections reaching layer 1 (e.g., Medalla and Barbas 2006). Puelles et al. (2024) noticed that all MCx sectors are relatively poor in parvalbumin expressing neurons, which possibly bespeaks of singular interneuronal circuitry properties. Moreover, the intrinsic mesocortical ring connections relate to a poorly myelinated condition of the local white matter (Palomero-Gallagher and Zilles 2004, 2015), which encompasses dorsomedially the classically identified cingular and angular bundles (present results).

**Fig. 14:**
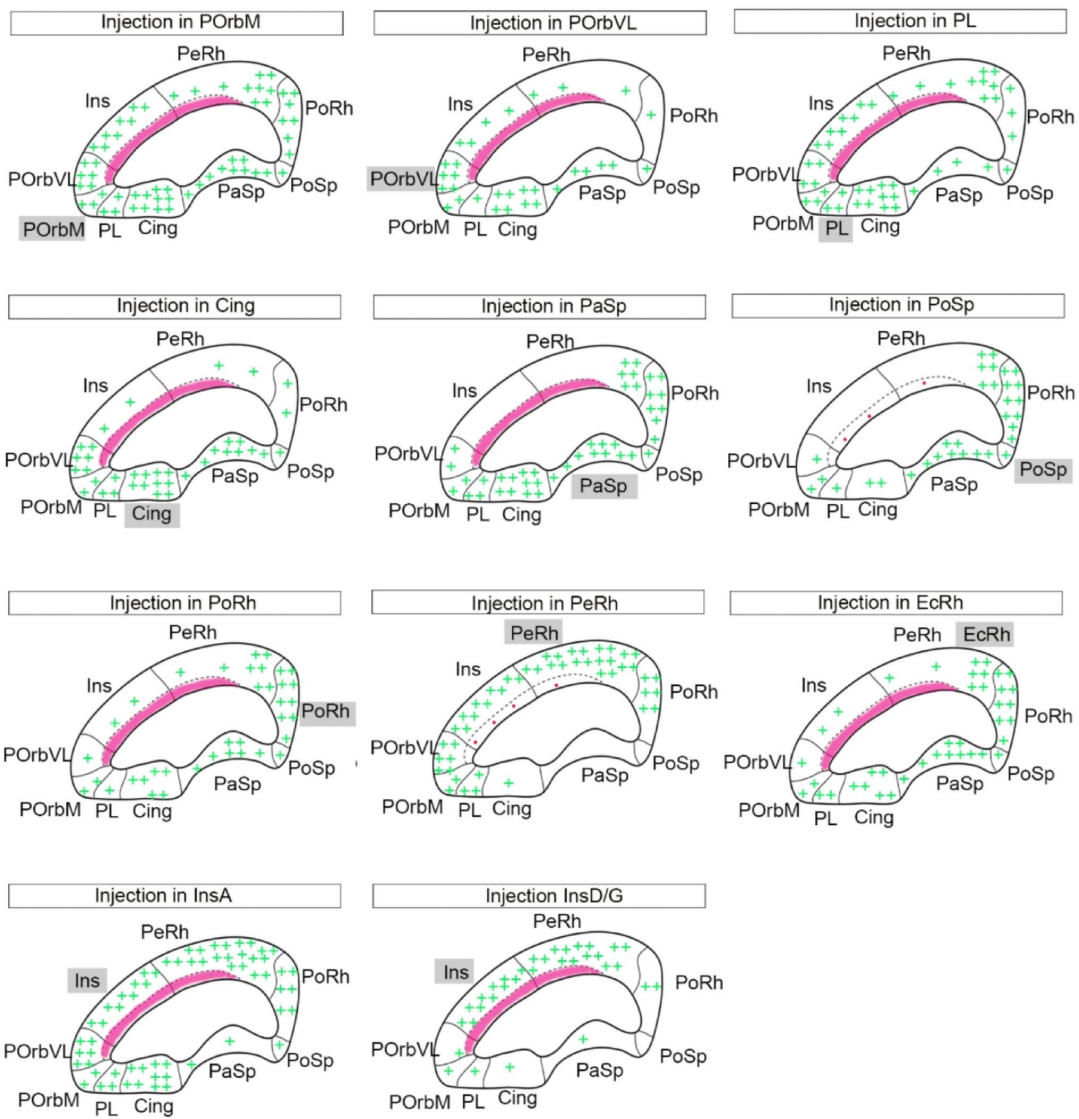
Summary of the internal connectivity of the mesocortical ring. Schema of the observed projections of individual ring sectors on thedifferent sectors of the mesocortical ring, distinguishing also those onthe claustrum in the POrbVL, Ins and PeRh sectors.

The crossed connections course through diverse places of the corpus callosum closest to their origins. Interestingly, the corpus callosum apparently carries 5%-17% unmyelinated axons both in the macaque and human (Lamantia and Rakic 1990; Aboitiz 1992). The functional signification of this unmyelinated component remains speculative so far (Innocenti et al. 2017) but might be illuminated if there is a correlation with limbic mesocortical origins. Exceptionally, the Ins sector shows crossed projections through the anterior commissure, a tract that also carries some poorly myelinated fibers. The intrinsic mesocortical ‘circular’ (ring-shaped) connective system underlined by our results may underpin bilateral limbic cortical integrative functions different from those sustained in parallel by means of limbic mesocortical connections with isocortex, allocortex, amygdala and subpallium (or descending connections with hypothalamic, thalamic, midbrain and brainstem targets). Recent data have suggested that interareal cortical connectivity in the mouse shows a hyperdense pattern compared to other mammals such as the marmoset and the macaque (Magrou et al. 2024). A widespread dense connectivity in the mouse was related to rapid emotional response, in contrast to the less dense modular network detected in the marmoset and macaque, potentially implicated in more complex cognitive processes. Present strictly mesocortical results in the mouse did not evidence exactly the widespread connectivity pattern described previously for other cortical areas (Magrou et al. 2024). Indeed, comparison with the mesocortex of primates should be highly interesting in order to evaluate any differences in connective density and their functional implications.

### Connections of individual mesocortical sectors

As stated above, the literature recorded hitherto a number of studies on the connectivity of some particular mesocortical sectors (mainly the cingulate, insular or orbital domains; reviews in Vogt 2009; Rolls 2014, 2019a,b; Rolls et al. 2020a) but did not elaborate a global picture of the whole mesocortical ring system. Present results obtained from a total of 61 ABMCA experiments strongly confirm the hypothesis that a system of intrinsic bilateral intralimbic connections does exist, with predominant ipsilateral effects.

We find that limbic cortices located in the medial regions of the ring, such as Cing and PaSp, project mainly onto neighboring medial cortices, rostralwards and caudalwards; conversely, cortices located in lateral areas of the ring, such as the Ins and PeRh regions, project similarly mainly onto lateral regions of the ring. A similar sectorial pattern occurs rostrally between the POrb domains of the ring and nearby rostromedial and rostrolateral ring sectors. Limbic cortices located caudally in the ring, such as PoSp and PoRh, project onto caudal sectors of the ring, including some caudomedial and caudolateral domains. These two sectors have a relatively low number of relevant experiments in the Allen database. In the only case that could be selected for PoSp, conditioned by the particularly small size of this area, the marker volume was below the optimal range desired. This implies that the projections from this locus may be more extensive than is described; in this case only the positive data are acceptable from a qualitative viewpoint but not the negative ones.

A summary of the global mesocortical intrinsic connectivity pattern is represented in Fig. 14. The clearcut overlaps existing between these partial connection patterns indicate that signals may be routinely transmitted sequentially around the whole ring, in both directions (clockwise and counter-clockwise). Furthermore, the POrbM cortex singularly receives input from all ring sectors and projects back onto all of them. The PL cortex exhibits a similar wide projection pattern as POrbM, but has weaker projections to the PaSp, PoSp, and PoRh cortices than POrbM.

### Insights into functional structure of the mesocortical limbic ring

In order to assess the potential global functional activity of the mesocortical ring suggested by these data it should be kept in mind that each mesocortical sector probably receives differential inputs from the neighboring associative domains of the isocortex and may produce also selective patterns of feedback into isocortex to intervene in attention, motivation, evaluation of experience and other aspects including consciousness. Simultaneously each limbic sector may feed forward its partial local state into parahippocampal allocortex, apart of relaying a copy to the other parts of the limbic cortical ring to which it connects (and systematically to the POrbM sector). From our present viewpoint, this pattern of corticocortical connections is known only fragmentarily, since solid data exist mainly for the best studied areas (POrb, Cing, and Ins), and equivalent data still need to be determined for the remaining ring sectors, which may have their own specificities. In order to obtain a better picture of the reaction of the whole limbic ring to the flow of evaluated experience and behavioral planning the less-known limbic areas need to be examined in similar detail and their precise number and identity must be determined. Potential sectorial molecular differences may serve to characterize them.

The intrinsic ring-formed mesocortical connectivity underlined in this report (topped by the hierarchically overlying activity of the POrbM area) may represent circulation of collateral copies of diverse specific analyses being performed in each sector of the ring, in a sort of multiple limbic re-entry mechanism that modulates in an integrated contextual manner the output at each of the partial sectors. This may be a mechanism that directs emotional attention to particular sets of data. The possible nature of the POrbM sector as a hierarchically superior level of the limbic structure might allow it to function as a selector of potentially diverse modes of work of the whole system. This may lead to a functionally multifactorial cohesive global structure of limbic evaluation of experience and behavioral plans, which may be interpreted differentially in every discrete sector of the ring, though in obeyance to orbitomedial modulation.

Different sectors of the mesolimbic ring have been related on the basis of stimulation studies and analysis of cortical pathology to the evaluation of specific stimuli (external or internal), and in some cases these algorithms have been linked to the functionality of other sectors. For example, the medial orbitofrontal cortex in humans has been linked to evaluation of reward, whereas the lateral orbitofrontal cortex appears associated to evaluation of non-reward and punishment (revised in Rolls et al. 2020b). Recent data emphasize the role of the ventrolateral orbital cortex in chronic pain and comorbid depression (Sheng et al. 2026).

Whether these functional differences reflect specifically limbic mesocortical functions (rather than prefrontal isocortical ones) is presently still unclear. There is the difficulty that the comparative correspondence of rodent and human ‘orbitofrontal’ areas is ambiguous as regards precise localization (delimitation; see e.g. van de Werd 2013). The conventional human ‘orbitofrontal’ cortex contemplated in the work of Rolls (*loc.cit*.) may refer in practice to a mixture of mesocortex and isocortex, when not just to some portion of human frontal isocortex, since habitually a strict distinction of mesocortical territories is not found in the literature. This problem may be addressed now purposefully using comparative genoarchitectonics. In the same work (Rolls et al. 2020a), connections of the orbitofrontal cortex with the insula are described. The latter is held to be an interoceptive area related with processing of emotions such as fear and disgust (Gogolla 2017; Klein et al. 2021). This suggests an exceptional primary sensory nature for one of the sectors of the mesocortical limbic ring, unless the conventional insular cortex also represents a mixture of mesocortical and isocortical domains. This doubt can again be checked in principle by application of several of the novel selective mesocortical gene markers.

Prelimbic cortex was implicated in fear memory and is connected to other limbic areas (Dixsaut and Gräff 2022; see their Figs. 3C for data on the PoSp; their Figs. 3G and O for PaSp; their Figs. 3K and O for PeRh and CL; and their Fig. 3W for Ins and CL). A claustro-prelimbic circuit was related to depressive disorders (Wang et al. 2023). Primate Area 25, which may correspond to PL in the mouse, was implicated in affective processes, and an analysis of its projection pattern relates this area with posterior orbitofrontal, anterior Cing and PeRh cortices (Joyce and Barbas 2018). PeRh and PoRh were described previously as interconnected areas (Burwell and Amaral 1998; Heimer-McGinn et al. 2017), and both were related with fear conditioning (Corodimas and LeDoux 1995; Bucci et al. 2000; Burwell et al. 2004; Heimer-McGinn et al. 2017; Meir et al. 2021). The prevalence of fear in many of these sectorial results (as well as in amygdala studies; see LeDoux 1996) suggests that this reactive aspect may be a global function mode of the whole ring/amygdala complex rather than a local function. So far, all these limbic brain areas have been analyzed from a local point of view, and their potential to act together, integrated by re-entry in a global limbic circuit, has not been considered at all.

Present results raise the possibility that the singularities in molecular profile of the mesocortical limbic ring as a whole (Puelles et al. 2024; Puelles and Garcia-Calero 2026 in press) may reflect some global integrative aspects of the processing of emotions and motivations that differ fundamentally from the analytic algorithm characteristic of the isocortex as a whole. The POrbM area may represent a hierarchically particularly relevant locus for integrating and modulating overall evaluative conclusions of the limbic ring. It might be even speculated that one integrative consequence of this functional system operating since early infancy might be the *Self* construct that underlies consciousness and intentional behaviour (Eccles 1989; LeDoux 1996; Damasio 1999, 2003; Edelman and Tononi 2000; Llinás 2001; Edelman 2004; Dehaene 2014). In this sense, Chanes and Barrett (2016) interestingly postulated that the limbic cortices participate in the emergence of a unified conscious experience, due to their connectivity and their position in the cortical hierarchy emphasized by the structural model of the cortex (i.e. Garcia-Cabezas et al. 2019). Chanes and Barrett (2016) obviously did not contemplate our ulterior variant molecular definition of the limbic cortex or mesocortex (Puelles et al. 2024; Puelles and García-Calero, in press) and thus included (conventionally) the parahippocampal gyrus and the temporal pole in the limbic cortex. We nevertheless coincide on this insight, which we apply specifically to our mesocortical limbic ring, without negating in principle the possible implication of allocortical components.

We think that such a construct is likely to have essential ontogenetic and evolutionary roots in an increasing limbic subjective appreciation and consequent memory of reality, obviously in collaboration with the more objective perceptual analysis of the thalamo-isocortical system. Behavioral success may use efficiently sectorial limbic analyses of particular stimuli but also may depend strongly on a global limbic assessment of the present, past and future experience under the intentional *Self* perspective (this borders the philosophical problem of free will).

### Where is the human mesocortex homologous to the mouse PaSp/PoSp domains?

A major new challenge is to translate these results to humans. It seems reasonable to assume that a limbic mesocortex exists also in the human. Its relative size within the cortex and its sectorial subdivisions may have increased allometrically in proportion to the more complex social life of primates and hominids (implying a richer and subtler emotional capacity). The classic posterior orbitary, prelimbic, cingulate, insular, perirhinal and postrhinal sectors of the ring are well know and have been object of numerous studies of various sorts, including fragmentary analysis of connectivity.

The novel PaSp and PoSp sectors introduced in the mouse MCx in place of the rejected classic retrosplenial cortex (Puelles et al. 2024) remain to be explored in the human brain. This conclusion was based on their shared molecular profile with the rest of limbic areas of the ring and is also consistent with parallel data of Palomero-Gallagher and Zilles (2004, 2015) on myelination and kainate receptor distribution. The classic ReSp was found to lack the limbic molecular profile and was reclassified as parahippocampal in nature (Puelles et al. 2024). The literature already suggests which human cortical areas may correspond to the mouse PaSP/PoSp, namely the so-called posterior cingulate domain of the interhemispheric cortex that surrounds dorsally the ReSp area. That is, the topological position remains identical (PaSp lies adjacent to ReSp), while the topography changes from a location at the dorsomedial convexity of the mouse cortex to the human interhemispheric surface (Vogt 2014). This change is clearly due to the larger surface growth of the cortex in primates and in the human, which pushes the PaSp/PoSp homolog areas medialwards.

### Role of POrbM in psychopathy

Interestingly, our data point out a possible superior hierarchic significance of the POrbM cortex within the mouse limbic ring. A similar, largely medial ‘orbitofrontal’ cortical region is emphasized as well in primate and human studies of emotion (Rolls 2014, 2015; Rolls et al. 2020a). Unfortunately, as mentioned above, these approaches to functional analysis do not distinguish yet methodologically what exactly is truly *limbic* (term understood by us as implying a molecularly defined mesocortical region of the limbic ring) and what represents instead specialized frontal *isocortex* (i.e., anterior orbitary and prefrontal isocortex). We hope to drive home thar present data jointly with the molecular ones of Puelles et al. (2024) suggest the convenience for clarity of distinguishing strictly the theoretically different functions performed by these two distinct types of cortex, irrespective of how deeply their mutual connections may be intermeshed.

Interestingly, we found in the book of Zald and Rauch (2006) on the orbitofrontal cortex various data suggesting that the personality disorder identified as *psychopathy* (also known as *pseudopsychopathy*, when caused by postnatal lesions, rather than congenital causes), has a causal background in the medial ‘orbitofrontal’ cortex (Koenigs and Tranel (2006). Psychopathy is characterized by a lack of empathy and antisocial behavior, suggesting complex (global) malfunction of the limbic system. In addition, it has been reported that patients with orbitofrontal damage often acquire psychopathic personalities (Eslinger and Damasio 1985; Barrash et al. 2000, 2022). When lesions in the orbitofrontal cortex are acquired at an early stage of life, antisocial behavioral traits are more pronounced (Taber-Thomas et al. 2014; Sanchez-Navarro et al. 2014; Anderson et al. 1999, 2000). These results suggest a role of ‘orbitofrontal’ cortex in both acquisition and execution of social/emotional knowledge. Ulterior studies also have detected in such psychopathic pathology low gray matter density in the orbitofrontal, cingulate and insular cortices, which also show abnormal activity patterns when viewing violent scenes (Decety et al. 2013; Nummenmaa et al. 2021; Myznikov 2024). Our present results indicate that the medial posterior orbitary cortex (i.e., what we suggest is the limbic part of the ‘orbitofrontal complex’) is hierarchically situated above the other sectors of the mesolimbic ring, and therefore, may play a key role in the healthy emotional evaluative functions of the individual that allows an equilibrated social behavior.

## Supplementary Information

Below is the link to the electronic supplementary material.

Supplementary Material 1

## Data Availability

No datasets were generated or analysed during the current study.

## Abbreviations

ac: Anterior commissure
ACAd: Anterior cingulate area, dorsal part, Allen Brain Atlas Nomenclature
ACAv: Anterior cingulate area, ventral part, Allen Brain Atlas Nomenclature
Acb: Nucleus accumbens
AI: Agranular insular area, Allen Brain Atlas Nomenclature
cc: Corpus callosum
cg: Cingulum
Cing: Cingulate cortex
CL: Claustrum
EcRh: Ectorhinal cortex
ECT: Ectorhinal cortex, Allen Brain Atlas Nomenclature
ERh: Entorhinal cortex
gcc: Genu of the corpus callosum
ICxFrA: Isocortex, frontal associative area
ICxM: Isocortex, motor area
ICxS: Isocortex, somestesic area
ICxV: Isocortex, visual area
ILA: Infralimbic area, Allen Brain Atlas Nomenclature
Ins: Insular cortex
ORBl: Orbital area, lateral part, Allen Brain Atlas Nomenclature
ORBm: Orbital area, medial part, Allen Brain Atlas Nomenclature
ORBvl: Orbital area, ventrolateral part, Allen Brain Atlas Nomenclature
PaSp: Parasplenial cortex
PeRh: Perirhinal cortex
PeRhA: Perirhinal cortex, anterior part
PeRhC: Perirhinal cortex, anterior part
PERI: Perirhinal cortex, Allen Brain Atlas Nomenclature
PL: Prelimbic cortex
POrbM: Posterior orbitary cortex, medial part
POrbVL: Posterior orbitary cortex, ventrolateral part
PoRh: Postrhinal cortex
PoSp: Postsplenial cortex
ReSp: Retrosplenial cortex
RSPagl: Retrosplenial area, lateral agranular part, Allen Brain Atlas Nomenclature
RSPv: Retrosplenial area, ventral part, Allen Brain Atlas Nomenclature
Se: Septum
St: Striatum
VISC: Visceral area, Allen Brain Atlas Nomenclature
VISpl: Posterolateral visual area, Allen Brain Atlas Nomenclature
VISpm: Posteromedial visual area, Allen Brain Atlas Nomenclature
VISpor: Postrhinal area, Allen Brain Atlas Nomenclatur
