# Supplementary material for: Internal connectivity of the mouse mesocortical ring and functional implications
Source: Brain Struct Funct. 2026 May 8;231(5):63. doi: 10.1007/s00429-026-03110-y (PMC13156105; doi:10.1007/s00429-026-03110-y)
Supplement: Supplementary file 1 — Supplementary Material 1 [file 429_2026_3110_MOESM1_ESM.docx]

**Supplementary Information**

**Title: Internal connectivity of the mouse mesocortical ring and functional implications**

Luis Puelles^1,2^ and Elena Garcia-Calero^1,2^

^1^Departamento de Anatomía Humana y Psicobiología, Facultad de Medicina, Universidad de Murcia, Campus de Ciencias de la Salud, 30120 Murcia, España.

^2^Instituto Murciano de Investigación Biomédica (IMIB), 30120 Murcia, España.

*Corresponding author:

[e-mail:ecalero@um.es](mailto:e-mail:ecalero@um.es)

**S1**

**
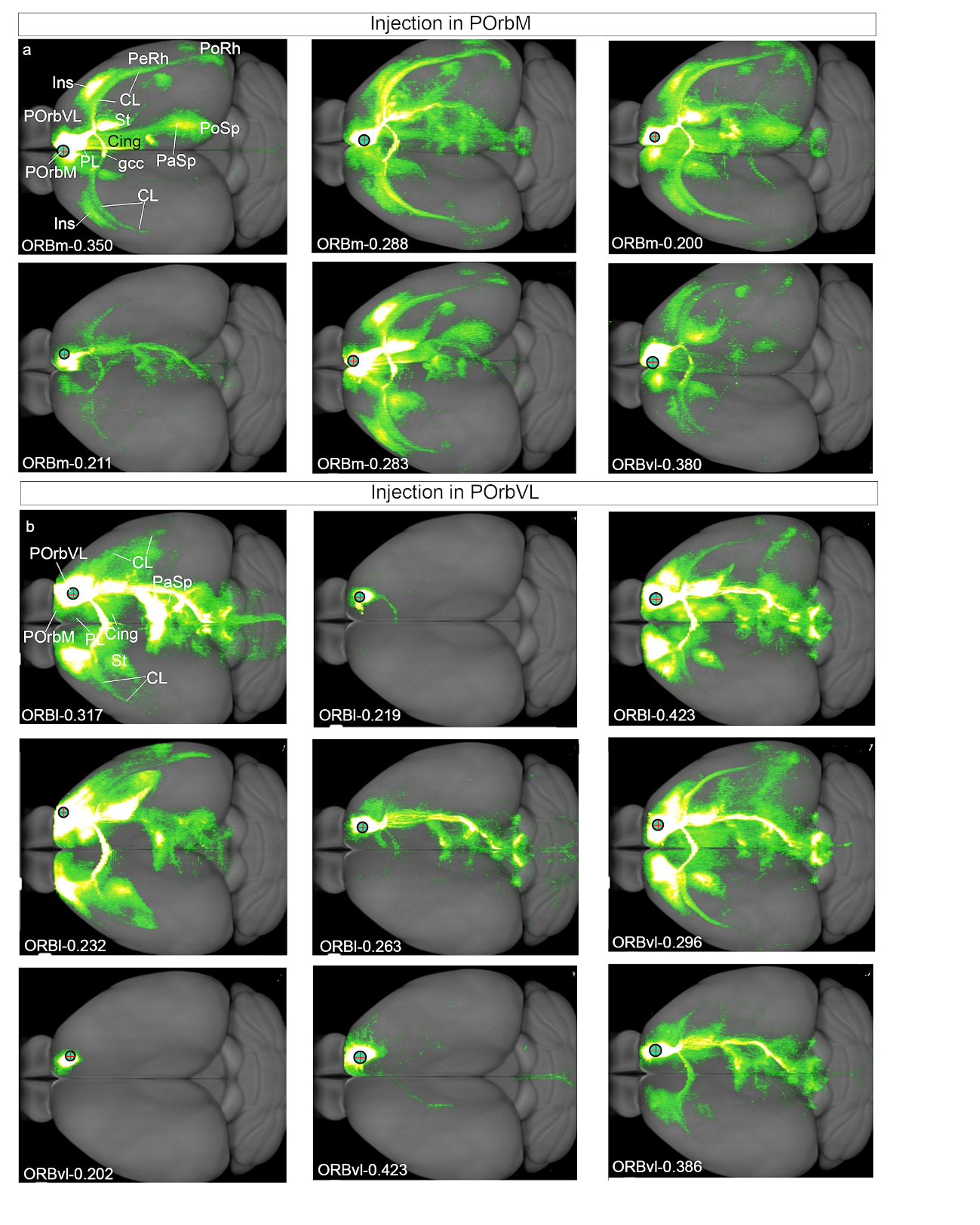
**

**S1. Density maps of injections in POrbM and POrbVL**

(a) Projection density maps of the cases injected in POrbM according to Table 1, obtained from the Allen Brain database (<https://brain-map.org/our-research/connectivity>). Case ORBvl-0.380 was reclassified as POrbM due to its relative caudomedial position in the orbital cortex (compare with cases in POrbVL).

(b) Projection density maps of the cases injected in POrbVL according to Table 1, obtained from the Allen Brain database (<https://brain-map.org/our-research/connectivity>). Vertical flip in cases: ORBm-0.288, ORBm-0.200, ORBvl-0.380. Cases ORBl-0.219, ORBvl-0.202 and ORBvl-0.423 showed low degree of tracer diffusion, but some labelled sectors were observed in the ring.

**S2**

**
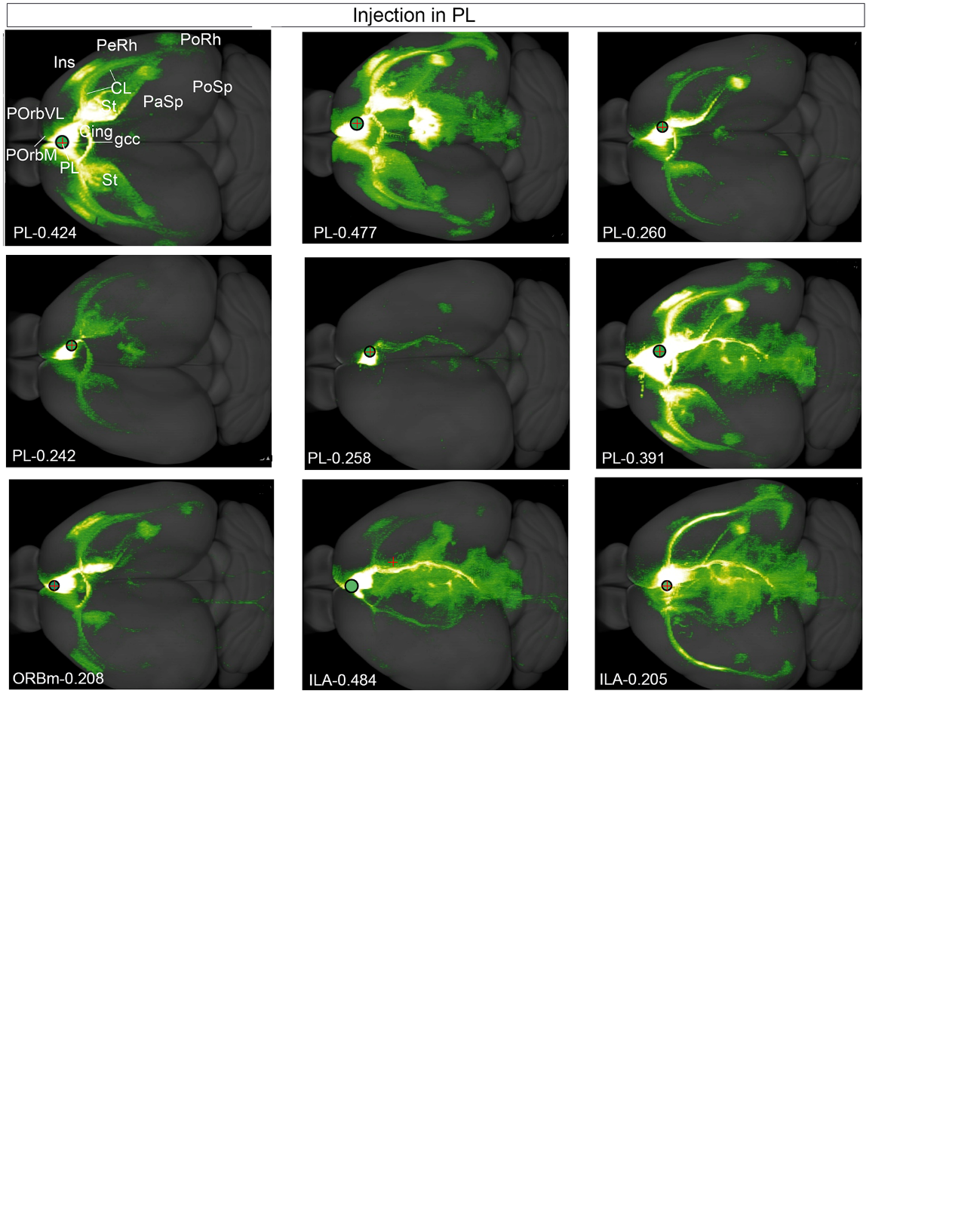
**

**S2. Density maps of injections in PL**

Projection density maps of the cases injected in PL according to Table 1, obtained from the Allen Brain database (<https://brain-map.org/our-research/connectivity>). Case ORBm-0.208 was reclassified as PL due to its more caudal position in the ring, comparable to other PL cases. Vertical flip in cases: PL-0.477, PL-0.242.

**S3**

**
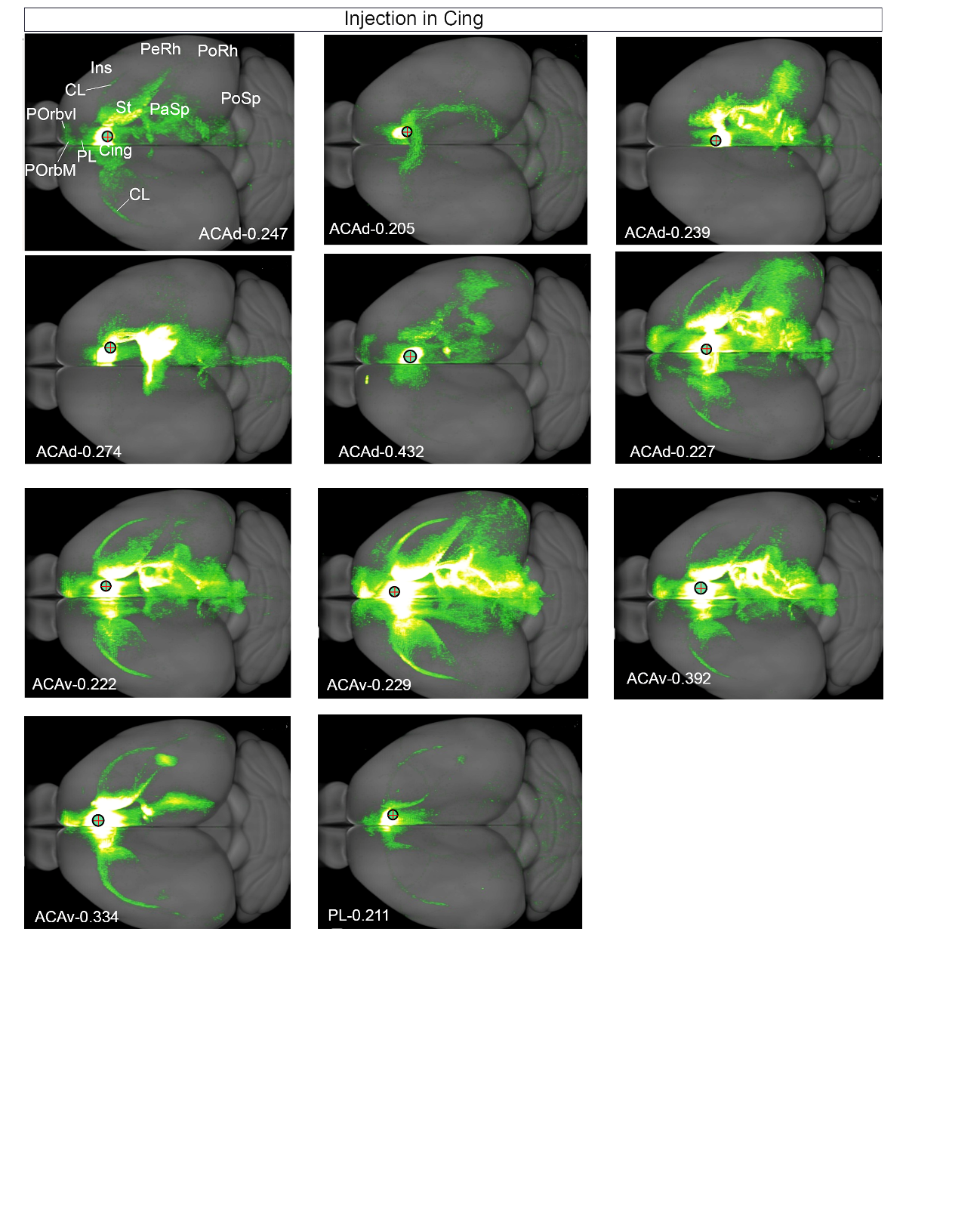
**

**S3. Density maps of injections in Cing**

Projection density maps of the cases injected in Cing according to Table 1, obtained from the Allen Brain database (<https://brain-map.org/our-research/connectivity>). Case PL-0.211 was reclassified as Cing due to its relative position in the ring. Vertical flip in case ACAd-0.227.

**S4**

**
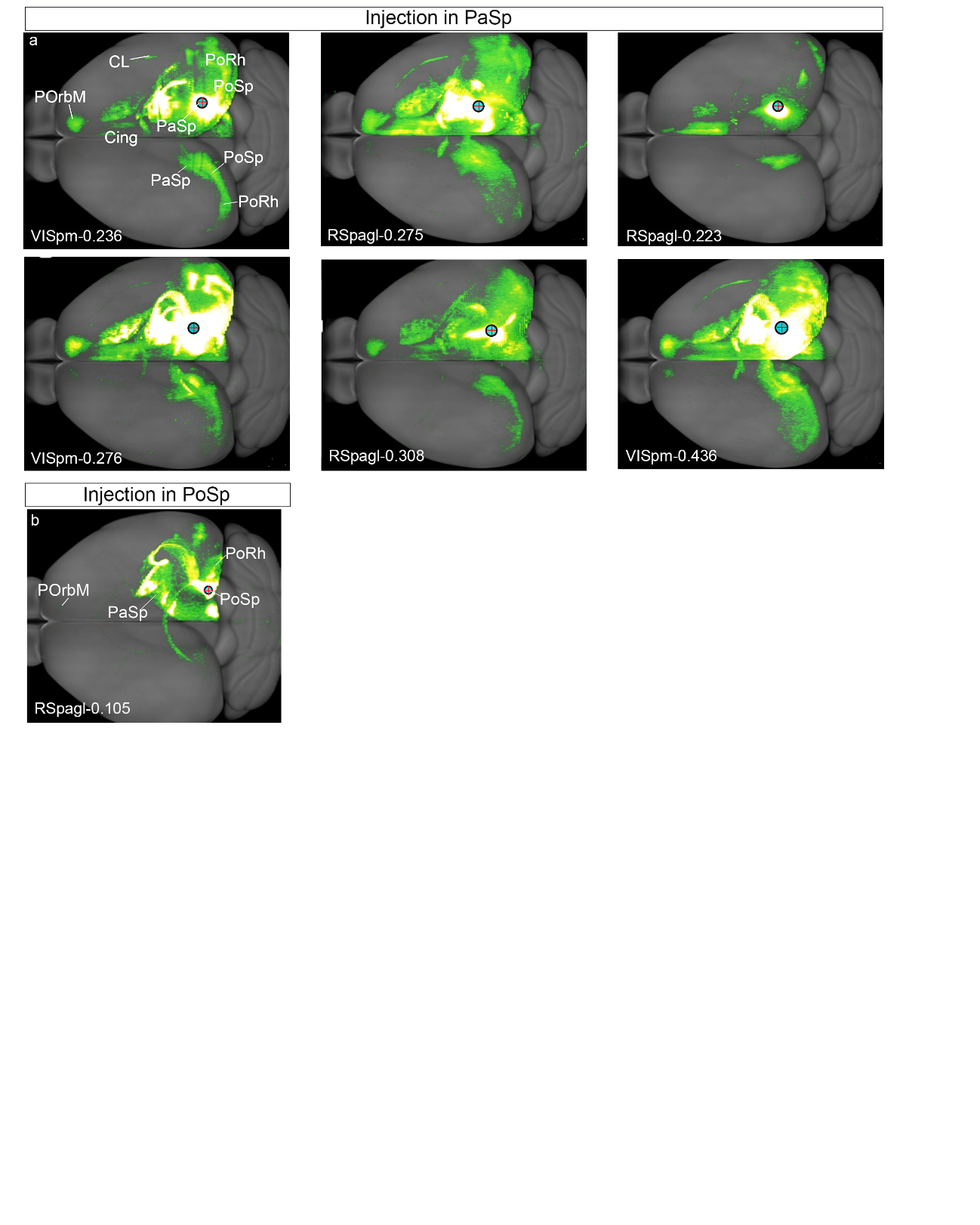
**

**S4. Density maps of injections in PaSp and PoSp**

(a) Projection density maps of the cases injected in PaSp according to Table 1, obtained from the Allen Brain database (<https://brain-map.org/our-research/connectivity>). (b) Projection density map of the case injected in PoSp according to Table 1, obtained from the Allen Brain database (<https://brain-map.org/our-research/connectivity>). Vertical flip in cases: RSpagl-0.308, RSpagl-0.275, RSpagl-0.223, VISpm-0.276, VISpm-0.236, VISpm-0.436.

**S5**


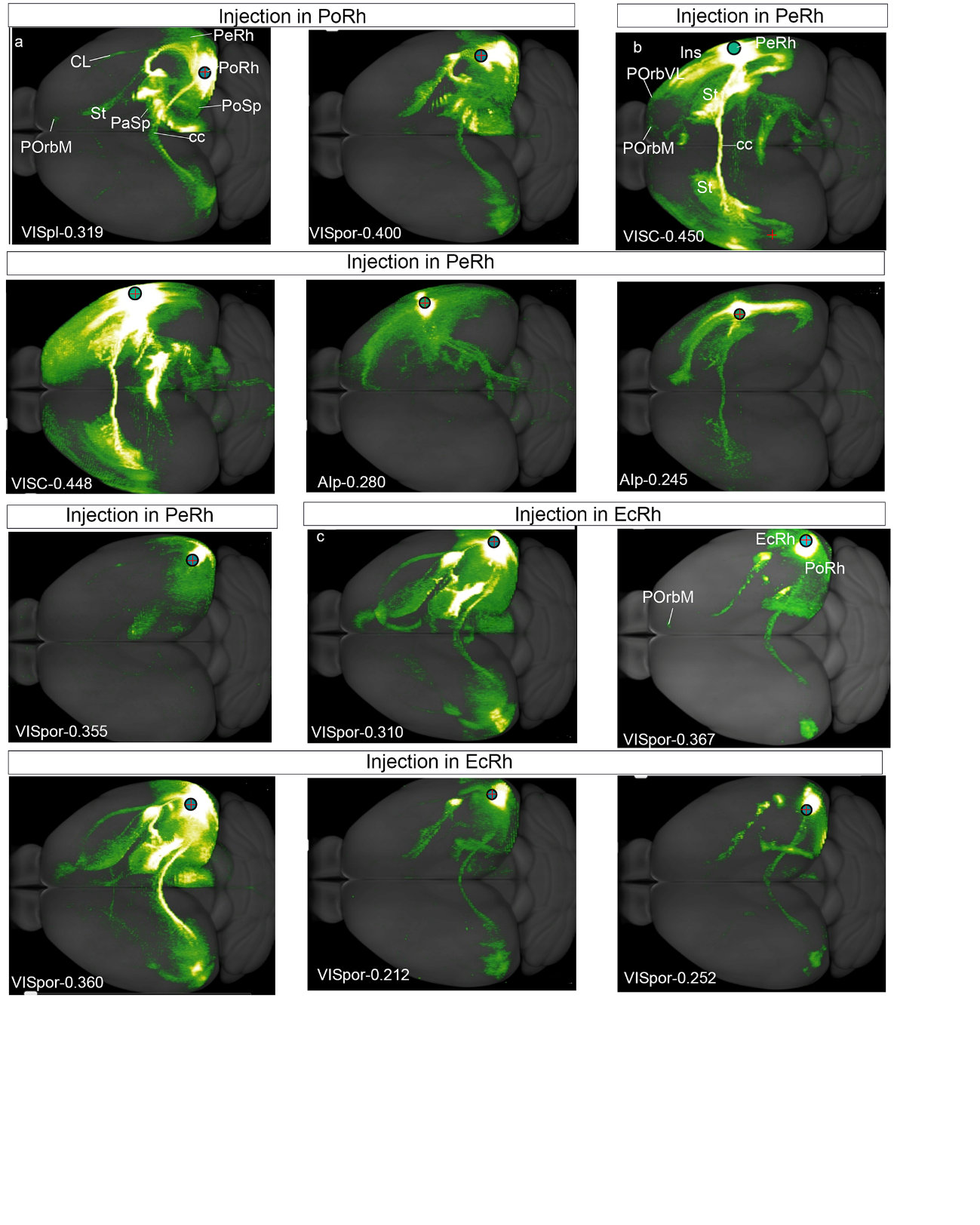


**S5. Density maps of injections in PoRh, EcRh and PeRh**

(a) Projection density maps of the cases injected in PoRh according to Table 1, obtained from the Allen Brain database (<https://brain-map.org/our-research/connectivity>).  (b) Projection density map of the case injected in PeRh according to Table 1, obtained from the Allen Brain database (<https://brain-map.org/our-research/connectivity>).  Cases AIp-0.280 and AIp-0.245 were reclassified as PeRh due to their position in the ring caudal to the level of the anterior commissure (c) Projection density maps of the cases injected in EcRh, obtained from the Allen Brain database (<https://brain-map.org/our-research/connectivity>).  Several VISpor cases were reclassified as PeRh (VISpor-0.355) or EcRh (VISpor-0.310, 0.367, 0.360, 0.212, 0.252) due to their position rostrally to PoRh, either in the agranular PeRh or the disgranular/granular EcRh cortices. Vertical flip in cases: VISpl-0.319, VISpor-0.400, VISpor-0.310, VISpor-0.367, VISpor-0.212, VISpor-0.252.

**S6**

**
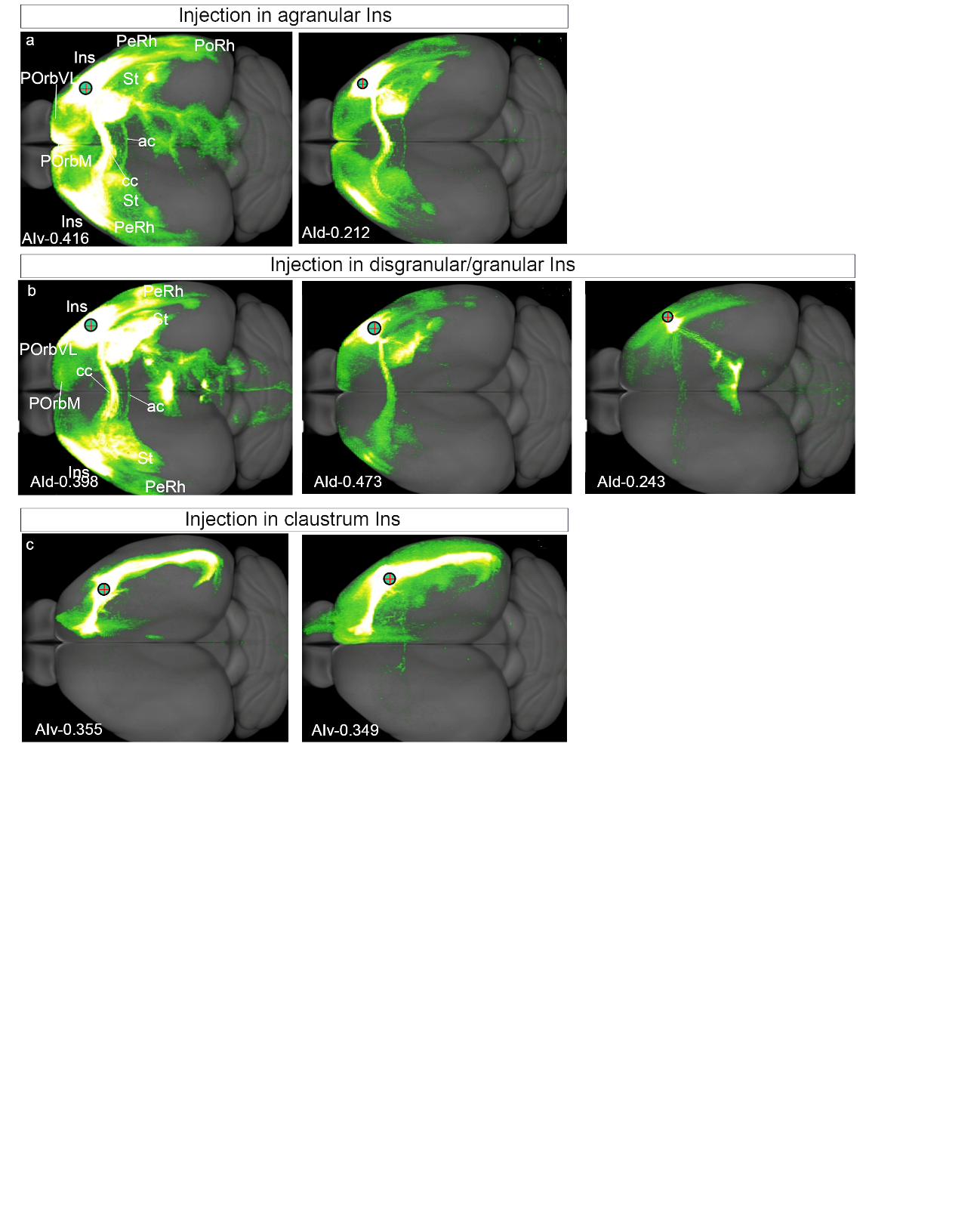
**

**S6. Density maps of injections in Ins agranular, disgranular/granular and claustral Ins**

(a) Projection density maps of the cases injected in agranular Ins according to Table 1, obtained from the Allen Brain database (<https://brain-map.org/our-research/connectivity>). (b) Projection density map of the case injected in disgranular/granular Ins according to Table 1, obtained from the Allen Brain database (<https://brain-map.org/our-research/connectivity>). (c) Projection density maps of the cases injected in insular claustrum, obtained from the Allen Brain database (<https://brain-map.org/our-research/connectivity>).
